# Critical Analysis of Particle Detection Artifacts in Synaptosome Flow Cytometry

**DOI:** 10.1523/ENEURO.0009-19.2019

**Published:** 2019-06-04

**Authors:** Benjamin D. Hobson, Peter A. Sims

**Affiliations:** 1 Department of Systems Biology, Columbia University Irving Medical Center, New York, NY 10032; 2 Department of Biochemistry and Molecular Biophysics, Columbia University Irving Medical Center, New York, NY 10032; 3 Medical Scientist Training Program, Columbia University Irving Medical Center, New York, NY 10032; 4 Sulzberger Columbia Genome Center, Columbia University Irving Medical Center, New York, NY 10032

**Keywords:** flow cytometry, synaptosomes

## Abstract

Flow cytometry and fluorescence-activated sorting are powerful techniques that hold great promise for studying heterogeneous populations of submicron particles such as synaptosomes, but many technical challenges arise in these experiments. To date, most flow cytometry studies of synaptosomes have relied on particle detection using forward scatter (FSC) measurements and size estimation with polystyrene (PS) bead standards. However, these practices have serious limitations, and special care must be taken to overcome the poor sensitivity of conventional flow cytometers in the analysis of submicron particles. Technical artifacts can confound these experiments, especially the detection of multiple particles as a single event. Here, we compared analysis of P2 crude synaptosomal preparations from murine forebrain on multiple flow cytometers using both FSC-triggered and fluorescence-triggered detection. We implemented multicolor fluorescent dye-based assays to quantify coincident particle detection and aggregation, and we assessed the false colocalization of antigens in immunostaining analyses. Our results demonstrate that fluorescence triggering and proper dilution can control for coincident particle detection, but not particle aggregation. We confirmed previous studies showing that FSC-based size estimation with PS beads underestimates biological particle size, and we identified pervasive aggregation in the FSC range analyzed in most synaptosome flow cytometry studies. We found that analyzing P2 samples in sucrose/EDTA/tris (SET) buffer reduces aggregation compared to PBS, but does not completely eliminate the presence of aggregates, especially in immunostaining experiments. Our study highlights challenges and pitfalls in synaptosome flow cytometry and provides a methodological framework for future studies.

## Significance Statement

Synaptosomes are an invaluable model for synaptic biology, but these synaptic particles are traditionally analyzed in bulk preparations rather than at the level of single particles. Although flow cytometry is a powerful technique for high-throughput particle analysis, submicron particles present unique challenges. Here, the authors investigate key elements of synaptosome flow cytometry experiments, especially those related to artifacts that confound the analysis of single synaptosomes. They identify aggregation as especially problematic and implement methods to minimize its impact on flow cytometry analysis.

## Introduction

Synaptosomes are synaptic particles consisting of resealed presynaptic nerve terminals that often remain bound to postsynaptic elements (Whittaker, 1993). These structures were originally isolated by homogenization of brain tissue in isotonic sucrose (Gray and Whittaker, 1962), and have been further purified using a variety of filtration and density gradient centrifugation procedures (Hollingsworth et al., 1985; Dunkley et al., 1986). Synaptosomes retain functional properties such as membrane potential and depolarization-induced neurotransmitter release, making them key model systems for fundamental synaptic biology (Whittaker, 1993). However, studies of synaptosomes in bulk are limited by the purity of the preparations, which contain a mixture of synapse types (e.g., glutamatergic, GABAergic, etc.) as well as contaminating neuronal and glial membranes. In principle, high-throughput purification and analysis of single synaptosomes is a powerful tool for addressing the incredible heterogeneity of the billions of synapses in mammalian brains.

Flow cytometry employs a pressurized fluidic system to pass suspensions of cells or particles through an optical flow cell, where scattered light and fluorescence measurements are collected for each particle. Analysis of submicron particles on conventional flow cytometers faces a variety of limitations and pitfalls due to the limited sensitivity of these instruments (Nolan, 2015). Event detection in flow cytometers, known as triggering, occurs when a particle passing through the flow cell causes the trigger parameter to rise above a manually set threshold. The trigger parameter, usually forward scatter (FSC), must have a minimum threshold set as to avoid electronic and buffer noise, but this limits detection of submicron particles. It is generally accepted that conventional flow cytometers have a lower limit of FSC-triggered detection around 300- to 500-nm PS beads (van der Pol et al., 2013), but this does not correspond to the size of biological particles that can be detected (Nolan, 2015; Lannigan et al., 2016). FSC intensity for submicron particles depends on many factors besides particle size, especially refractive index, which is generally 1.59–1.61 for PS beads, 1.40–1.46 for silica beads, and ranges from 1.33 to 1.40 for cell-derived particles (Nolan, 2015). Biological particles therefore scatter light ∼10-fold less efficiently than PS beads (Chandler et al., 2011), and direct comparison of PS bead FSC intensity has been shown to underestimate the size of biological particles (Chandler et al., 2011; van der Pol et al., 2012; Simonsen, 2016).

In addition to difficulties with size estimation using light scatter, numerous reports have identified particle detection artifacts in submicron flow cytometry. Particles below the trigger threshold can be detected when analyzed at high concentrations, a phenomenon known as coincidence or “swarm detection” (van der Pol et al., 2012; Nolan and Stoner, 2013). While coincidence is caused by the simultaneous presence of multiple, single particles in the path of the laser, others have reported detection of single events comprised of multiple, aggregated submicron particles (Erdbrügger et al., 2014). Although these artifacts have been recognized in the cell-derived microparticle community (Nolan, 2015; Lannigan et al., 2016), most flow cytometry studies of synaptosomes have not addressed triggering or detection artifacts (Gylys et al., 2000; Fein et al., 2008; Postupna et al., 2014; Prieto et al., 2017). Despite claims that single synaptosomes can be detected by FSC triggering and identified apart from contaminating particles based solely on FSC signal (Gylys et al., 2004; Gylys and Bilousova, 2017), this has been a point of recent controversy (see comments on Prieto et al., 2017). In contrast to these studies, Biesemann and Herzog found that sorting of FSC-triggered events bearing genetically encoded vesicular glutamate transporter 1 (VGLUT1) fluorescence yielded samples contaminated with GABAergic synaptosomes and myelin (Biesemann, 2010; his Results 3.4, Fig. 14, pp 86–87). Instead, fluorescence triggering and sorting events below the FSC trigger threshold yielded a VGLUT1 synaptosomal sample of unprecedented purity (Biesemann et al., 2014). The flow cytometry protocols published by these groups are clearly at odds in terms of the best practices to ensure detection, analysis, and sorting of single synaptosomes (Gylys and Bilousova, 2017; Luquet et al., 2017).

Here, we present a critical flow cytometry analysis of P2 crude synaptosome preparations on two cytometers, the BD Influx and BD LSRFortessa. We investigated several experimental aspects of synaptosome flow cytometry, including: (1) FSC-triggered and fluorescence-triggered particle detection, (2) FSC-based size estimation with PS and silica bead standards, (3) range of particle concentration, (4) coincidence and aggregation as causes of false double-positive events, (5) sucrose and PBS buffers for sample preparation and acquisition, and (6) false colocalization of antigens in immunostained samples. Our study highlights technical challenges and identifies methods to minimize their impact on experimental results.

## Materials and Methods

### Animals

Male C57BL/6J mice (6–10 weeks old) were used in all experiments. Mice were housed on a 12/12 h light/dark cycle with food and water available ad libitum. All animal procedures were performed in accordance with the Columbia University Institutional Animal Care and Use Committee and followed National Institutes of Health guidelines.

### P1 crude nuclei and P2 crude synaptosome preparations

Preparation of the P2 crude synaptosome fraction was performed using standard procedures (Gray and Whittaker, 1962). Mice were sacrificed by cervical dislocation, after which forebrains were rapidly dissected and placed in 10 volumes of ice-cold buffer consisting of 0.32 M sucrose, 4 mM HEPES (pH 7.4), and protease inhibitors (cOmplete EDTA-free protease inhibitor, Roche). Tissue was homogenized on ice in a glass-glass dounce homogenizer with 10 gentle strokes of loose and tight clearance pestles. All subsequent purification steps were performed on ice or at 4°C unless otherwise specified. The homogenate was centrifuged at 1000 × *g* (Eppendorf 5424R) for 10 min to remove nuclei and cellular debris, yielding a P1 pellet and an S1 supernatant.

A crude nuclei preparation was prepared for flow cytometry according to established procedures (Krishnaswami et al., 2016). The P1 pellet was resuspended in ice cold buffer containing 250 mM sucrose, 25 mM KCl, 5 mM MgCl_2_, 10 mM Tris (pH 7.4), 1 µM DTT, 0.1% Triton X-100, and 1 µM Hoechst 33342 and homogenized again with 10 strokes of the tight pestle to facilitate release of nuclei. The homogenate was rotated for 15 min at 4°C, filtered through a 40-µm cell strainer cap, and centrifuged at 500 × *g* (Eppendorf 5424R) for 5 min to yield a crude nuclear pellet.

The S1 supernatant was further centrifuged at 10,000 × *g* (Eppendorf 5424R) for 20 min to obtain the crude synaptosome pellet (P2). P2 pellets were cryopreserved by resuspension in 4 mM HEPES/0.32 M sucrose buffer + 5% DMSO and slowly frozen to –80°C using an isopropanol freezing container. Frozen synaptosomes were used within two months. This protocol, when combined with rapid thawing at 37°C on the day of the experiment, has been shown to preserve synaptosome function and morphology (Gleitz et al., 1993; Daniel et al., 2012). After thawing, all experiments were conducted with either PBS (137 mM NaCl, 2.7 mM KCl, 8 mM Na_2_HPO_4_, and 2 mM KH_2_PO_4_) or sucrose/EDTA/tris (SET) buffer (320 mM sucrose, 5 mM Tris, and 1 mM EDTA).

### Flow cytometry instrumentation and setup

All flow cytometry data acquisition was conducted using the instrument software FACSDiva (BD Biosciences). All flow cytometry data analysis including gating, quantification, and generation of density plots/histograms was performed using FCS Express 6 (De Novo Software). Number of events, % of all events, and channel statistics (median, geometric mean, SD, etc.) for all gates were exported using “Batch Export” for further statistical analysis.

All data acquisition on LSRFortessa (hereafter Fortessa, BD Biosciences) was conducted using the lowest possible sample pressure settings. Optical configuration employed and fluorophores detected in these channels are summarized in Table 1.

**Table 1.** Optical configuration of the LSRFortessa

LSRFortessa optics				
Laser	Band pass filter	Dichroic filter	Detector name	Fluorophores used in this study
405 nm	450/50	Blank	Pacific Blue	Violet amine-reactive dye
488 nm	488/10	Blank	SSC	
488 nm	530/30	505LP	FITC	Alexa Fluor 488, Calcein AM
488 nm	710/50	685LP	PerCP-Cy5.5	FM4-64
561 nm	582/12	Blank	PE	Alexa Fluor 555, Calcein red
633 nm	670/30	Blank	APC	Alexa Fluor 647, MitoTracker Deep Red FM

The powers of the lasers in the Fortessa are 405 nm 100 mW, 488 nm 50 mW, 561 nm 100 mW, 640 nm 100 mW. Detector voltages underwent minor fluctuations over the course of these studies to maintain comparable sample fluorescence and FSC/SSC values for the fluorescent microspheres, which were run at the beginning of each experiment. Approximate detector voltages were as follows: FSC (300), SSC (265), Pacific Blue (407), FITC (588), PerCP-Cy5.5 (500), PE (511), and APC (537).

The BD Influx (hereafter Influx, BD Biosciences) was operating using a 100-μm nozzle at 11.1 psi. The sample pressure differential was kept as low as possible. The frequency was set at 25.8 kHz, piezo amplitude between 5 and 10. Optical configuration employed and fluorophores detected in these channels are summarized in Table 2.

**Table 2.** Optical configuration of the Influx

Influx optics			
Laser	Band pass filter	Detector name	Fluorophores used in this study
405 nm	460/50	BV421	Hoechst 33342, Violet amine-reactive dye
488 nm	488/10	SSC	
488 nm	530/40	FITC	Alexa Fluor 488, Calcein AM
488 nm	692/40	PerCP-Cy5.5	FM4-64
561 nm	589/29	PE	Alexa Fluor 555, Calcein red
638 nm	670/30	APC	Alexa Fluor 647, MitoTracker Deep Red FM

The power of the lasers in the Influx are 405 nm 100 mW, 488 nm 200 mW, 561 nm 120 mW, 640 nm 120 mW. Detector voltages underwent minor fluctuations over the course of these studies to maintain comparable sample fluorescence and FSC/SSC values for the bead standards. Approximate detector voltages were as follows: FSC (22.70), SSC (25.77), BV421 (52.55), FITC (44.51), PerCP-Cy5.5 (41.68), PE (63.65), APC (54.78).

In the annual preventive and maintenance conducted by the BD engineer, a quality control (QC) is run with the six-peak rainbow beads. In the daily setup, the QC setting is retrieved and the daily alignment is compared with the QC setting. The complete overlay of the six peaks with the QC setting indicates the proper alignment of the laser. Drop delay is done with the drop delay beads and the accudrop camera, a step which calculates the distance between the laser interrogation point and the charging point.

Daily setup also includes running a mixture of fluorescent PS bead standards (Bangs Labs, Inc.) and a mixture of non-fluorescent silica and fluorescent PS bead standards (Apogee Flow Systems). To ensure reproducibility in FSC-positioning of samples across experiments, the positions of bead populations on FSC versus SSC and FSC versus FITC (green fluorescence detector for PS beads) plots are maintained within tight gates stored in the QC workspace. Only occasionally did this require minor adjustments to detector voltages.

### Flow cytometry gating

Gating of bead populations was performed using density plots of FSC versus SSC and FSC versus FITC (green fluorescence detector for PS beads). Gating of all fluorescent channels was performed using a polygon gate on a bivariate density plot of FSC versus fluorophore. Boolean logic gating was used to assess all intersections of positive versus negative for each fluorophore (e.g., double-negative, calcein AM+/calcein red-, calcein AM-/calcein red+, double-positive). Where applicable, hierarchical gating was used to assess all intersections of positive versus negative for each fluorophore within each FSC gate.

### FSC-triggered and FM4-64-triggered detection

On both the Influx and Fortessa, the FSC voltages were set to place 500-nm PS beads just left of center on the FSC axis, near 10^2^ on the Influx and near 10^3^ on the Fortessa. This enabled the beads to be detected just above the FSC noise threshold while also allowing 800-nm PS and 1300-nm Sil beads to be collected on scale. Critically, this also allowed for a significant amount of floor space on the FSC axis below the FSC noise threshold, which became accessible on fluorescence triggering with FM4-64.

For FSC triggering on the Influx and Fortessa, the FSC trigger threshold was first lowered to the minimum value on the instrument while running clean PBS to define the range of electronic, optical, buffer, and all other sources of FSC noise. After collecting ∼10,000 events, the FSC voltage was raised to the minimum level just above the FSC noise threshold such that event rates were <10 events/s while running clean PBS. Using the configurations described above, the FSC-trigger threshold was generally 250–300 on the Fortessa and generally 0.9–1.10 on the Influx.

FM4-64 fluorescent triggering was implemented on the Influx and Fortessa using a procedure similar to FSC triggering. FM4-64 was obtained from ThermoFisher, reconstituted at 100 µg/µl, and diluted into PBS or sucrose buffer at 1.5 µg/ml. This concentration was previously shown to be effective for fluorescence triggering of synaptosomes (Biesemann et al., 2014; Luquet et al., 2017). We found optimal detection of FM4-64 fluorescence in the PerCP-Cy5.5 channel and used this channel for triggering on the Influx and Fortessa. As with FSC triggering, the fluorescence trigger threshold was first lowered to a minimum while running clean buffer + FM4-64 to define the noise range. The fluorescent trigger threshold was raised to the minimum level above the noise range such that event rates were <10 events/s while running buffer + FM4-64. Using the configurations described above, the FM-trigger threshold was generally 120–150 on the Fortessa and generally 0.35–0.40 on the Influx.

### Fluorescence-activated sorting

For sorting on the Influx, populations of interest were gated using the FACSDiva software. For bead double-positive and double calcein-positive sorting, the event rate was <5000 events/s. The sort was conducted using 1.0 Drop Pure mode. At least 20,000 events were sorted directly into FACS tubes, which were then used to reanalyze the sorted samples. Reanalysis was conducted <15 min after sorting using the exact same cytometer settings, and at least 1500 events were re-analyzed.

### Multicolor labeling and mixing assays

Cryopreserved P2 samples were rapidly thawed at 37°C, washed in PBS, and centrifuged at 10,000 × *g* (Eppendorf 5424R) for 5 min at 4°C. Pellets were resuspended in PBS and equally divided into separate tubes for single dye labeling. Synaptosomes were incubated at room temperature in PBS supplemented with one of the following dyes: calcein AM for 15–30 min at a final concentration of 1 µM, calcein red-orange AM (hereafter calcein red) for 15–30 min at a final concentration of 1.9 µM, violet amine-reactive dye for 30 min (1:1000 from 50-µl stock, as per manufacturer’s guidelines), or MitoTracker Deep Red FM for 30 min at a final concentration of 500 nM. Synaptosomes were returned to ice and subsequently washed and re-centrifuged twice with ice-cold PBS to remove residual dye. All samples were maintained on ice and protected from light after labeling. After the second wash, pellets were resuspended in equal volumes of PBS + 1.5 µg/ml FM4-64 and mixed in equal volumes to generate mixtures of single color-labeled synaptosomes. In most experiments, these mixtures were re-centrifuged and resuspended before passing the suspension through a 40-µm cell strainer cap before flow cytometry. In time lapse experiments, individual samples were passed through a 40-µm cell strainer cap and mixed immediately before flow cytometry. For experiments employing different buffer combinations, the above protocol was identical, with SET used in place of PBS as indicated in Figure 6*G*: PBS represents the standard assay; PBS-SET represents the standard assay but with the mixing, 3rd wash, and flow cytometry conducted in SET instead of PBS; and SET employs SET instead of PBS for all steps. For fluorescence mixture assays, we observed some variability in the calcein-labeling efficiency across samples. Since only fluorescent particles are eligible to report on coincidence or aggregation, we normalize each fluorescent event type (i.e., single-positive/double-positive) to all fluorescent events within each sample.

### Immunostaining

Crude nuclear pellets were resuspended in blocking buffer (PBS, pH 7.4 with 5% BSA) supplemented with 1 µM Hoechst 33342 and blocked on ice for 15 min. Primary antibody staining was conducted in blocking buffer at 4°C on a rotator for 30 min. Samples were washed with blocking buffer, centrifuged at 500 × *g* (Eppendorf 5424R) for 5 min and resuspended in blocking buffer with goat anti-mouse Alexa Fluor 488. Secondary staining was 4°C on a rotator for 20 min. Samples were again washed with blocking buffer plus 1 µM Hoechst 33342 and centrifuged at 500 × *g* (Eppendorf 5424R) for 5 min, following resuspension in PBS for flow cytometry.

P2 crude synaptosomes were briefly fixed in 4% paraformaldehyde (PFA) in PBS, pH 7.4 for 15 min at room temperature. After washing in blocking buffer to quench residual PFA, synaptosomes were centrifuged at 10,000 × *g* (Eppendorf 5424R) for 5 min at 4°C. The P2 pellet was resuspended in blocking buffer + 0.1% Tween 20 at room temperature for 30 min before addition of primary antibodies. Primary antibody staining was conducted at room temperature for 30 min, followed by two washes in PBS with centrifugation and resuspension. Pellets were resuspended in blocking buffer + 0.1% Tween 20 with secondary antibodies and stained for 20 min at room temperature. Samples were washed twice with PBS, and final samples were resuspended in PBS + 1.5 µg/ml FM4-64 for flow cytometry. For the PBS-SET condition, the final wash and flow cytometry were conducted in SET buffer.

### Absolute particle count measurements

P2 samples from dilution series (Extended Data Fig. 2-1*C*) or from repeated centrifugation experiments (Fig. 8; Extended Data Fig. 8-1) were diluted to a precise final volume (200–500 µl) in PBS+FM or SET+FM. A precise volume of resuspended CountBright absolute counting beads was added to each sample. Counting beads were run alone to confirm that they could be gated apart from FM4-64-triggered P2 events, and were gated based on SSC and blue fluorescence (BV421 channel). Particle concentration was calculated for each sample as per the manufacturer’s guidelines using the number of FM4-64-triggered P2 events, count bead events, bead count of the lot (beads/µl), and bead/sample volumes. For repeated centrifugation experiments (all using indicated relative centrifugal force (× *g*) on Eppendorf 5424R), absolute particle counts were calculated based on the total sample volume, and total particle counts in each fraction were determined by back-calculation using the aliquot and sample volumes.

### Alexa Fluor 488 MESF calibration

Quantum Alexa Fluor 488 MESF calibration beads were run on the Influx at the same time and under identical conditions (sample pressure, detector voltages, etc.) as the immunostained samples. MESF calibration beads (7 µm) could be identified based on SSC with only a slight reduction (∼10%) in the SSC voltage for this experiment. The five calibration beads (blank and MESF beads 1–4) were run individually to confirm separation by the green fluorescence detector (FITC channel). Beads were first gated based on SSC and then based on green fluorescence histograms (Extended Data Fig. 7-1*E*) using full width at half-maximum gating as recommended by the manufacturer. The median fluorescence intensity of the calibration beads was used to fit the MESF calibration curve (Extended Data Fig. 7-1*E*) using the QuickCal V.2.3 template from the manufacturer (Bangs Laboratories, Inc.). The calibration curve was used to calculate the median fluorescence intensity of indirect immunofluorescence [Ms anti-vesicular GABA transporter (VGAT) + anti-mouse Alexa Fluor 488] in MESF for subsets of gated events (Extended Data Fig. 7-1*F*). Numerical data are available in Extended Data Figure 7-3.

### Beads, chemicals, and antibodies

Polystyrene (PS) bead standards (200, 500, and 800 nm in diameter) with refractive index ɳ = 1.59 and Yellow-Green fluorescent label were obtained from Polysciences Inc. (Submicron Bead Calibration kit, catalog number BLI832, Bangs Laboratories Inc.). A bead standard mixture of Yellow-Green fluorescent PS beads with refractive index ɳ = 1.59 (110 and 500 nm in diameter) and non-fluorescent silica beads with refractive index ɳ = 1.43 (180, 240, 300, 585, 880, and 1300 nm in diameter) was obtained from Apogee Flow Systems (product #1493). Unlabeled PS beads (2.0 µm in diameter) were obtained from Spherotech Inc. (catalog number PP-20-10). CountBright absolute counting beads were obtained from Invitrogen (catalog number C36950). Quantum Alexa Fluor 488 MESF calibration bead kit was obtained from Bangs Laboratories, Inc. (catalog number 488).

The following dyes were obtained from Invitrogen/Invitrogen: Hoechst 33342 (catalog number H3570), FM4-64 (catalog number T13320), calcein AM (catalog number C34852), calcein red-orange AM (catalog number C34851), LIVE/DEAD Fixable Violet amine-reactive dye (catalog number L34955), and MitoTracker Deep Red FM (catalog number M22426). Antibodies used in these studies and their concentrations are summarized in Table 3.

**Table 3.** List of antibodies

Primary antibody list						
Origin	Antigen		Concentration	Manufacturer	Product number	RRID
Mouse	NeuN		1:5,000	Millipore	MAB377	AB_2298772
Mouse	VGAT		1:400	Synaptic Systems	131 011	AB_887872
Guinea pig	VGLUT1		1:500	Synaptic Systems	135 304	AB_887878
Rabbit	VMAT2		1:500	Immunostar	20042	AB_10013884
Secondary antibody list						
Origin	Antigen	Fluorophore	Concentration	Manufacturer	Product number	RRID
Goat	Mouse IgG (H + L)	Alexa Fluor 488	1:500	Invitrogen	A-11001	AB_2534069
Goat	Guinea Pig IgG (H + L)	Alexa Fluor 555	1:500	Invitrogen	A-21435	AB_2535856
Goat	Rabbit IgG (H + L)	Alexa Fluor 647	1:500	Invitrogen	A-21244	AB_2535812

### Statistical analyses

Gated data exported from FCS Express 6 were further processed in Excel and in R. Where indicated, the robust SD (rSD) was calculated using FCS Express 6 software. All graphs were generated using the *ggplot2* package in R, except those in Figure 8 and Extended Data Figures 2-1*C*, 7-1*C–F*, 8-1*A–F*, which were generated in Excel. All statistical testing was conducted in R. Where indicated, a Welch’s *t* test was performed with the indicated contrasts (Figs. 5*D*, 6*D–F*; Extended Data Fig. 6-1*C*). Where indicated, a two-way ANOVA was performed using FSC Gate and Buffer as factors (Figs. 6*H*, 7*B*,*C*); degrees of freedom, *F* statistic, and *p* values for main effects as well as interaction are listed in the figure captions. A summary of all statistical tests is provided in the statistical table (Table 4).

**Table 4.** Statistical table

Line	Figure	Comparison	Data structure	Statistical test	Power	*p* value	*F/t* value
a	5*D*	Mix 0 min vs spin 0 min	Normal distribution	Two-sided Welch’s *t* test	0.9950	0.0072	*t*_(3.49)_ = –5.64
b	5*D*	Mix 40 min vs spin 40 min	Normal distribution	Two-sided Welch’s *t* test	0.8313	0.0046	*t*_(5.23)_ = –4.74
c	5*D*	Mix 75 min vs spin 75 min	Normal distribution	Two-sided Welch’s *t* test	0.8251	0.0103	*t*_(7.86)_ = –3.35
d	6*D*	FSC noise vs <500-nm PS	Normal distribution	Two-sided Student’s *t* test	0.6783	0.0260	*t*_(11.29)_ = –2.56
e	6*D*	<500-nm PS vs <880-nm sil	Normal distribution	Two-sided Welch’s *t* test	0.8487	0.0085	*t*_(11.62)_ = 3.16
f	6*D*	<880-nm sil vs <1300-nm sil	Normal distribution	Two-sided Welch’s *t* test	0.4452	0.0738	*t*_(14.71)_ = –1.92
g	6*D*	<1300-nm sil vs >1300-nm sil	Normal distribution	Two-sided Welch’s *t* test	0.7761	0.0124	*t*_(14.83)_ = 2.85
h	6*E*	FSC noise vs <500-nm PS	Normal distribution	Two-sided Welch’s *t* test	0.6953	0.0242	*t*_(10.95)_ = 2.61
i	6*E*	<500-nm PS vs <880-nm sil	Normal distribution	Two-sided Welch’s *t* test	0.8366	0.0092	*t*_(11.73)_ = –3.11
j	6*E*	<880-nm sil vs <1300-nm Sil	Normal distribution	Two-sided Welch’s *t* test	0.4428	0.0734	*t*_(15.73)_ = 1.92
k	6*E*	<1300-nm sil vs >1300-nm sil	Normal distribution	Two-sided Welch’s *t* test	0.7423	0.0142	*t*_(15.54)_ = –2.76
l	6*F*	FSC noise vs <500-nm PS	Normal distribution	Two-sided Welch’s *t* test	0.0911	0.5286	*t*_(2.00)_ = 0.76
m	6*F*	<500-nm PS vs <880-nm sil	Normal distribution	Two-sided Welch’s *t* test	0.9965	0.0229	*t*_(2.00)_ = –6.5
n	6*F*	<880-nm sil vs <1300-nm sil	Normal distribution	Two-sided Welch’s *t* test	0.2397	0.2468	*t*_(2.01)_ = 1.62
o	6*F*	<1300-nm sil vs >1300-nm sil	Normal distribution	Two-sided Welch’s *t* test	0.7653	0.0230	*t*_(3.99)_ = –3.59
p	6-1*C*	PBS vs PBS-SET	Normal distribution	Two-sided Welch’s *t* test	0.0666	0.6775	*t*_(14.11)_ = 0.425
q	6-1*C*	PBS-SET vs SET	Normal distribution	Two-sided Welch’s *t* test	0.2500	0.1779	*t*_(12.49)_ = 1.43
r	6-1*C*	PBS vs SET	Normal distribution	Two-sided Welch’s *t* test	0.5481	0.0417	*t*_(17.44)_ = 2.20
s	6*H*	Main effect; FSC gate	Normal distribution	Two-way ANOVA	0.9793	1.83E-13	*F*_(4,125)_ = 21.40
t	6*H*	Main effect; buffer	Normal distribution	Two-way ANOVA	0.5278	1.15E-06	*F*_(2,125)_ = 15.29
u	6*H*	Interaction: FSC gate × buffer	Normal distribution	Two-way ANOVA	0.2030	0.0043	*F*_(8,125)_ = 2.98
v	7*B*, VGAT	Main effect; FSC gate	Normal distribution	Two-way ANOVA	0.9753	2.84E-10	*F*_(4,50)_ = 21.21
w	7*B*, VGAT	Main effect; buffer	Normal distribution	Two-way ANOVA	0.0815	0.0610	*F*_(1,50)_ = 3.67
x	7*B*, VGAT	Interaction: FSC gate × buffer	Normal distribution	Two-way ANOVA	0.0526	0.8060	*F*_(4,50)_ = 0.40
y	7*B*, VGLUT1	Main effect; FSC gate	Normal distribution	Two-way ANOVA	0.9998	<2E-16	*F*_(4,50)_ = 68.31
z	7*B*, VGLUT1	Main effect; buffer	Normal distribution	Two-way ANOVA	0.0500	0.724	*F*_(1,50)_ = 0.13
a1	7*B*, VGLUT1	Interaction: FSC gate × buffer	Normal distribution	Two-way ANOVA	0.0504	0.956	*F*_(4,50)_ = 0.16
b1	7*B*, VMAT2	Main effect; FSC gate	Normal distribution	Two-way ANOVA	0.8516	4.67E-07	*F*_(4,50)_ = 12.35
c1	7*B*, VMAT2	Main effect; buffer	Normal distribution	Two-way ANOVA	0.0781	0.069	*F*_(1,50)_ = 3.46
d1	7*B*, VMAT2	Interaction: FSC gate × buffer	Normal distribution	Two-way ANOVA	0.0513	0.887	*F*_(4,50)_ = 0.28
e1	7*C*, single+	Main effect; FSC gate	Normal distribution	Two-way ANOVA	0.9756	2.71E-10	*F*_(4,50)_ = 21.27
f1	7*C*, single+	Main effect; buffer	Normal distribution	Two-way ANOVA	0.1074	0.029	*F*_(1,50)_ = 5.06
g1	7*C*, single+	Interaction: FSC gate × buffer	Normal distribution	Two-way ANOVA	0.0504	0.96	*F*_(4,50)_ = 0.16
h1	7*C*, double+	Main effect; FSC gate	Normal distribution	Two-way ANOVA	0.9876	1.68E-11	*F*_(4,50)_ = 25.33
i1	7*C*, double+	Main effect; buffer	Normal distribution	Two-way ANOVA	0.1335	0.0162	*F*_(1,50)_ = 6.20
j1	7*C*, double+	Interaction: FSC gate × buffer	Normal distribution	Two-way ANOVA	0.0530	0.7857	*F*_(4,50)_ = 0.43
k1	7*C*, triple+	Main effect; FSC gate	Normal distribution	Two-way ANOVA	0.6865	1.69E-05	*F*_(4,50)_ = 8.88
l1	7*C*, triple+	Main effect; buffer	Normal distribution	Two-way ANOVA	0.0544	0.255	*F*_(1,50)_ = 1.33
m1	7*C*, triple+	Interaction: FSC gate × buffer	Normal distribution	Two-way ANOVA	0.0507	0.938	*F*_(4,50)_ = 0.20

## Results

### FSC and FM4-64 triggering of crude synaptosome fraction P2 and comparison to bead standards

Conventional flow cytometers generally use FSC measurements for detection of cells, but the sensitivity of each instrument in FSC-triggered detection of submicron particles varies widely depending on the refractive index of the particles, the angle of collection for scattered light, detector sensitivity, and other factors (van der Pol et al., 2013). We used PS and silica beads to determine the relative sensitivity of two cytometers: a BD LSR Fortessa analyzer and a BD Influx sorter. We optimized FSC-threshold parameters on each instrument to maintain a mixture of PS and silica bead standards ranging 585–1300 nm in the dynamic range of detection, while simultaneously avoiding electronic and buffer noise.

**Figure 1.**
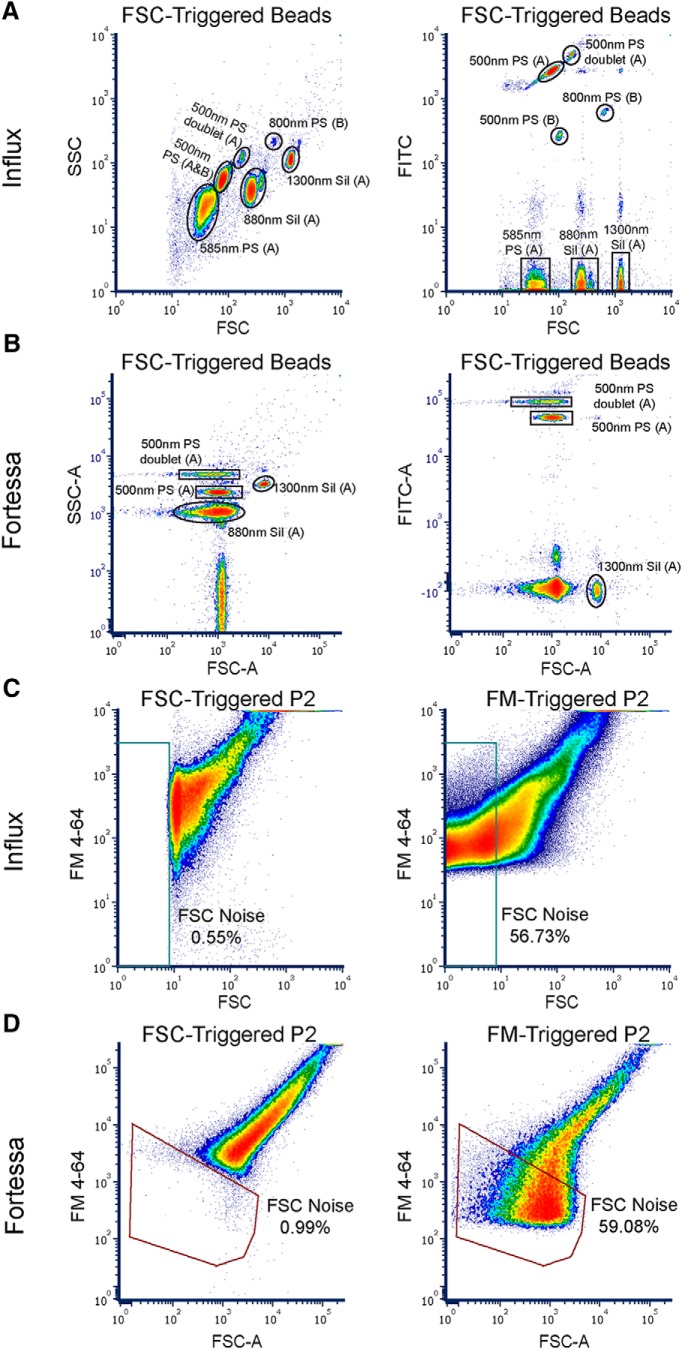
Determining detection sensitivity using bead standards and FSC versus FM triggering of P2 fraction. ***A***, Mixture of non-fluorescent silica/fluorescent PS beads (Apogee; A) and fluorescent PS beads (Bangs Labs; B) detected in FSC-trigger mode on the Influx. Forward and side scatter (left) FSC (right) and green fluorescence detector (FITC) allows fluorescent PS beads and bead doublets to be clearly distinguished from non-fluorescent silica beads. ***B***, Mixture of non-fluorescent silica/fluorescent PS beads (Apogee; A) detected in FSC-trigger mode on the Fortessa. Note that the Bangs Labs 500- and 800-nm fluorescent PS beads could not be run simultaneously with the Apogee mixture on the Fortessa due to poor FSC resolution. ***C***, FM4-64-stained P2 sample on the Influx. Left, FSC-trigger mode. Right, FM-trigger mode. FSC noise gate was set by running clean PBS. ***D***, FM4-64 stained P2 sample on the Fortessa. Left, FSC-trigger mode. Right, FM-trigger mode. FSC noise gate was set by running clean PBS. See Extended Data Figure 1-2 for median FSC and SSC values from gated bead populations. See also Extended Data Figure 1-1.

As shown in Figure 1*A*, the lowest FSC signal resolvable above the background noise on the Influx was the 585-nm silica bead. Cross-referencing the FSC signal with the green fluorescence signal allowed us to clearly identify PS beads apart from the non-fluorescent silica beads (Fig. 1*A*, right). As expected due to their higher refractive index, the FSC signals for PS beads were substantially higher than for similarly sized silica beads. We also identified events that appeared to be simultaneous detection of two 500-nm PS beads, which we confirmed by comparing their FSC and green fluorescence to the “singlet” gate as previously described (Extended Data Fig. 1-2; Stoner et al., 2016). Although the FSC and fluorescent signals appeared additive in the 500-nm PS doublet population (1.7-fold higher FSC, 1.8-fold higher green fluorescence), the FSC signal for these events still fell below the 880-nm silica beads. In contrast to the Influx, the Fortessa displayed poor FSC resolution when running the silica/PS bead mixture (Fig. 1*B*), which required separately running the individual PS beads [500- and 800-nm PS labeled with (B) in Influx plots]. The lowest FSC signal resolvable above the noise background on the Fortessa included 880-nm silica as well as 500-nm PS singlet and “doublet” bead populations, which could only be separated by SSC or green fluorescence intensity (Fig. 1*B*). A complete summary of FSC and SSC measurements for gated bead populations can be found in Extended Data Figure 1-2.

10.1523/ENEURO.0009-19.2019.ed1 Extended Data Figure 1-1 Comparison of FSC signal from neuronal nuclei and 0.5-, 0.8-, and 2.0-µm PS beads. We note that in these experiments, the FSC detector voltage had to be lowered in order to allow the 2.0-µm PS to fall within the detectable range; the FSC axis is therefore substantially different from all other plots in this study. ***A***, Mixture of fluorescent PS beads (Bangs Labs; B, or Spherotech; S) detected in FSC-trigger mode on the Influx (left) and SSC-trigger mode on the Fortessa (right). We note that the lowered FSC voltage on the Fortessa resulted in a significant amount of FSC noise. ***B***, Crude nuclei detected in FSC-trigger mode on the Influx. Left, Hoechst33342 fluorescent staining of DNA identifies nuclei apart from all other particles. Right, Gated nuclei are then assessed for immunofluorescence of NeuN to identify neuronal nuclei. ***C***, Overlay of 2.0-µm PS bead gate (left) and neuronal nuclei (right) using FSC trigger on the Influx. Download Extended Data 1, TIF file.

**Figure 2.**
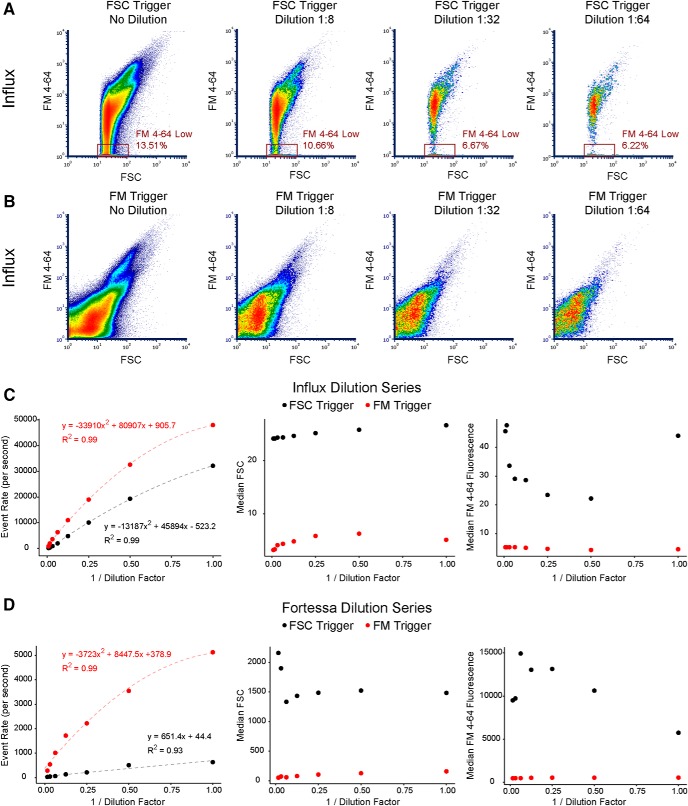
Dilution series define a range of linear particle detection in P2 samples. ***A***, Representative density plots of a P2 dilution series detected on the Influx in FSC-trigger mode. FM4-64 low gate was set arbitrarily to quantify disappearance of events in this region with dilution. ***B***, Representative density plots of same P2 dilution series as in ***A***, but detected on the Influx in FM-trigger mode. ***C***, Measurements from full P2 dilution series on the Influx. Left, Event rate data fit with a quadratic model. Middle, Median FSC of all detected events. Right, Median FM4-64 fluorescence of all detected events. ***D***, Measurements from full P2 dilution series on the Fortessa. Left, FM-trigger series fit with a quadratic model and FSC-trigger series fit with a linear model. Middle, Median FSC of all detected events. Right, Median FM4-64 fluorescence of all detected events. See also Extended Data Figure 2-1.

10.1523/ENEURO.0009-19.2019.f1-2 Extended Data Figure 1-2 FSC and SSC Measurements of PS and Silica Beads on Influx and Fortessa. Median FSC and SSC measurements with coefficient of variation (CV) for gated bead populations in Figure 1. Data are listed in order of ascending median FSC, which does not correspond to true size due to the refractive index mismatch between silica and PS beads. Download Figure 1-2, DOC file.

Having established FSC-triggering limits and relative bead positions on the instruments, we next sought to compare FSC and fluorescence triggering of P2 crude synaptosome (hereafter P2) samples. Despite contamination by a heterogeneous mixture of free mitochondria, myelin, and membranous debris, P2 samples are enriched with synaptosomes and have been widely employed in flow cytometry studies (Gylys et al., 2000; Fein et al., 2008; Postupna et al., 2014; Prieto et al., 2017). As previously described by Biesemann et al. (2014), FM4-64 is a styryl dye with favorable properties for fluorescence-triggered detection of synaptosomes. FM4-64 is minimally fluorescent in aqueous media but becomes intensely fluorescent on partitioning into membranes (Vida and Emr, 1995), and its far-red emission is efficiently excited by a 488nm laser, which is required for triggering on our cytometers. As shown in Figure 1*C*,*D*, FM triggering enabled highly sensitive detection of P2 samples compared to FSC triggering, with more than half of particles in the P2 sample below the FSC noise threshold on both cytometers. These results are consistent with the work of Biesemann (2010; his Results 3.5, Fig. 15, pp 87–90), who found ∼70% of particles in sucrose gradient synaptosome preparations were undetectable by FSC triggering on the BD FACS Aria.

Since previous studies have analyzed putative synaptosomal particles with FSC signals as high as 4.5-µm PS beads (Gylys et al., 2000), we sought to assess the size of biological particles in such an FSC range. In the absence of a refractive index mismatch between PS beads and biological particles, one would expect neuronal nuclei, which sediment in the P1 fraction, to exhibit significantly higher FSC than 2-µm PS beads due to their size. Although we had to lower the FSC voltage to place 0.5-, 0.8-, and 2-µm PS beads within the same dynamic range, 2.0-µm PS beads were clearly resolved from 0.5- to 0.8-µm PS beads by FSC on both the Influx and Fortessa (Extended Data Fig. 1-1*A*). We identified neuronal nuclei based on Hoechst 33342 intensity and NeuN immunofluorescence (Extended Data Fig. 1-1*B*) and found that they were adjacent to and partially overlapping with the FSC range defined by 2-µm PS beads on the Influx (Extended Data Fig. 1-1*C*). These results confirm that FSC-based sizing relative to PS beads underestimates the size of biological particles with lower refractive indices, and suggest that a majority of nuclei would be included in FSC gates containing 4.5-µm PS beads.

### Serial dilutions define a range of linear detection for FM4-64 triggering

It has previously been demonstrated that high concentrations of particles below the trigger threshold can be detected when they simultaneously occupy the focal point of illumination (van der Pol et al., 2012; Nolan and Stoner, 2013). This phenomenon of coincident detection (also known as “coincidence” or swarm detection) is thought to be particularly problematic for FSC-triggered detection of submicron particles (Nolan and Stoner, 2013). to define a linear range of particle detection and further compare FSC versus FM triggering, we conducted dilution series on both Influx and Fortessa. Compared to FM triggering, FSC-triggered P2 samples did not maintain a constant FSC versus FM4-64 profile on either the Influx or Fortessa (Fig. 2*A*,*B*; Extended Data Fig. 2-1). Specifically, the abundance of events near the FSC threshold with low FM4-64 fluorescence decreased with dilution, and the appearance of “noise” events (although <10 events/s) became more apparent with dilution on the Fortessa. On the Influx, quantification of the event rate across the dilution series revealed a sub-linear profile for both FSC and FM triggering, which was fit with high accuracy using a quadratic model (*R*^2^ = 0.99 for both series; Fig. 2*C*, left panel). FM triggering was similar on the Fortessa, but FSC triggering was better fit with a linear model (*R*^2^ = 0.93 for FSC and *R*^2^ = 0.99 for FM series; Fig. 2*D*, left panel). These results confirm that event rate saturates at high particle concentrations (Kormelink et al., 2016) and that FSC triggering underestimates the true event rate across the entire dilution series.

10.1523/ENEURO.0009-19.2019.f2-1 Extended Data Figure 2-1 Dilution series define a range of linear particle detection in P2 samples (Fortessa). ***A***, Representative density plots of a P2 dilution series detected on the Fortessa in FSC-trigger mode. FM4-64 low gate was set arbitrarily to quantify disappearance of events in this region with dilution. Although FSC-trigger threshold was set to reduce noise to below 10 events/s, at the low end of the dilution curve these events begin to comprise a significant fraction of collected data. ***B***, Representative density plots of same P2 dilution series as in ***A***, but detected on the Fortessa in FM-trigger mode. ***C***, Typical dilution series with FM4-64 triggering on the Influx, conducted with absolute counting beads. FM4-64 triggered event counts (excluding absolute counting beads) were used to determine particle concentration of each sample dilution. Event rate (left) and calculated particles/µl (right) are shown across the dilution series. Download Figure 2-1, TIF file.

Given that much of the P2 remains undetected by FSC triggering, we also wondered whether particle concentration might alter the FSC and FM fluorescence measurements. On the Influx, the median FSC remained relatively constant for both FSC and FM triggering (Fig. 2*C*, middle panel), while the median FM4-64 fluorescence varied dramatically for FSC triggering but not for FM triggering (Fig. 2*C*, right panel). On the Fortessa, both median FSC and median FM4-64 fluorescence varied dramatically across the dilution series for FSC triggering but not FM triggering (Fig. 2*D*, middle and right panels). The increase in median FM4-64 fluorescence with dilution seen in FSC-triggered samples seems related to the “FM4-64 low” events described above. Given the heterogeneous population of particles contained in P2 samples, we cannot make strong conclusions about the mechanisms underlying these effects. Nonetheless, it is clear that FM triggering is superior to FSC triggering in terms of the stability and accuracy of event rate, FM4-64 fluorescence, and FSC of P2 particles across the dilution series on both instruments. We conducted all further studies using only FM triggering and maintaining reasonably dilute samples so as to stay in the most linear range of the event rate dilution curves (fewer than 10,000 events/s on the Influx and fewer than ∼3000 events/s on the Fortessa; Fig. 2*C*,*D*, left panels). Since sample flow rates and event rates may vary across cytometers, we also measured the absolute particle concentration across a typical dilution series on the Influx using volumetric count beads (Extended Data Fig. 2-1*C*). We found that the most linear range of the event rate curve was for samples below ∼1000 particles/µL. A previous study on the Influx (Kormelink et al., 2016) showed that the trigger pulse baseline becomes continuously elevated at high event rates where coincidence is prominent, so we used a digital oscilloscope on the Influx to confirm that the trigger pulse remained at baseline under these conditions (data not shown).

### Development of a multicolor fluorescence assay for false colocalization

**Figure 3.**
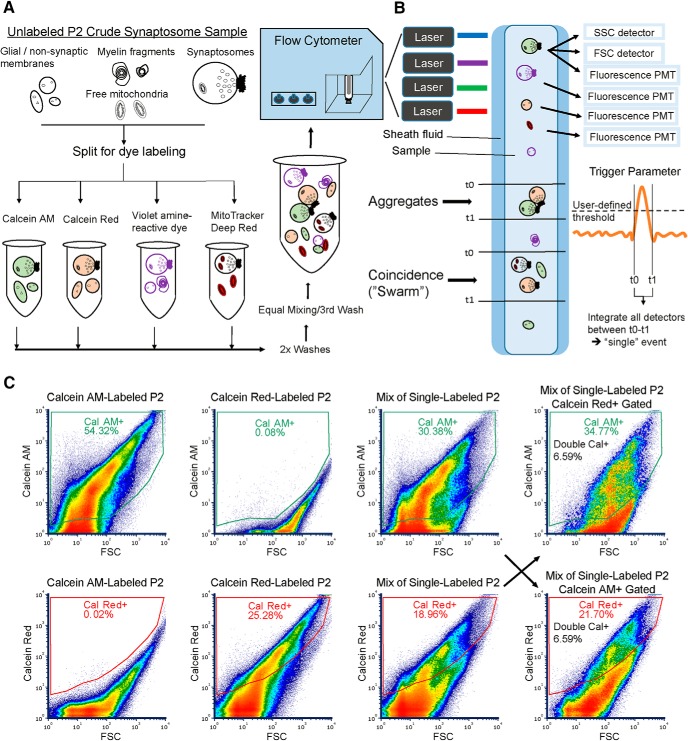
Experimental assay for measuring coincidence and aggregation. ***A***, Schematic representation of the P2 sample labeling workflow. A single sample is split into equal portions for single-color labeling, followed by two washes to remove residual dye. The samples are then mixed and washed a third time before flow cytometry. ***B***, Schematic representation of particle detection and artifacts in microparticle flow cytometry. Particles flow in the sample fluid, which is surrounded by sheath fluid. Note that the width of the sample stream is enlarged for illustrative purposes; we run our cytometers at the minimum sample pressure (and thus sample stream width) to ensure optimal hydrodynamic focusing. When the trigger parameter detector crosses a user-defined threshold, the time duration of this pulse is then integrated on all detectors to generate an event. Single events that are detection artifacts occur when multiple particles simultaneously occupy the focal point of laser illumination (coincidence) or are physically bound (aggregates). ***C***, Representative density plots of a P2 sample from the double-calcein assay on the Influx. Left, Calcein AM-labeled P2 sample shows no cross-emission in the calcein red detector. Mid-left, Calcein red-labeled P2 sample shows no cross-emission in the calcein AM detector. Mid-right, Mixture of single-labeled P2 samples is gated for calcein AM+ and calcein red+ particles, which are then cross-checked for the other fluorophore (diagonal arrows). Right, Calcein AM+ or calcein red+ gated particles are analyzed to quantify double calcein+ events. See also Extended Data Figure 3-1.

Although FM triggering and dilution should help to reduce coincident particle detection, another reported source of “false double-positive” events in submicron flow cytometry is the physical aggregation of multiple particles (Erdbrügger et al., 2014). Multiple microscopy studies have noted the presence of aggregated synaptosomes (Gray and Whittaker, 1962; Choi et al., 2009; Daniel et al., 2012), but conventional flow cytometry does not provide a means to verify whether events truly represent single particles. We sought to design a fluorescence-based flow cytometry assay to estimate false double-positive events in our P2 samples (Fig. 3).

10.1523/ENEURO.0009-19.2019.f3-1 Extended Data Figure 3-1 Lack of spectral overlap between dyes and lack of calcein dye transfer to unstained samples. ***A***, Matrix of representative density plots demonstrating lack of spectral overlap between any of the four dyes used in multicolor aggregation assays. Columns represent P2 samples single-labeled with the indicated dye, while rows indicate the measured fluorescence in the detector for the indicated dye. All samples were detected by FM triggering on the Influx. ***B***, A single P2 sample was split into equal aliquots, one of which was left unstained, while the other was truly doubled-labeled with calcein-AM and calcein red. The double-labeled sample was then sonicated in the equivalent volume and concentration of PBS and subsequently mixed with the unstained sample as in the standard multicolor aggregation assay. A small aliquot of the double-labeled sample (right) and the sonicated double-labeled/unstained mixture (left) were detected using FM triggering on the Influx. As shown, sonication fully disrupted all double-labeled particles, and none of the released calcein dye was acquired by the unstained sample. Download Figure 3-1, TIF file.

The assay workflow is similar to previously described “cell barcoding” flow cytometry methods (Krutzik and Nolan, 2006; Krutzik et al., 2011). A P2 sample is first split and single-labeled with spectrally separated, non-transferable fluorescent dyes. After washing, single-labeled samples are mixed before flow cytometry (Fig. 3*A*). Single particle events should therefore bear only one fluorescent label, while coincidences and aggregates derived from separate single-labeled samples will be positive for multiple fluorescent labels (Fig. 3*B*). Although the samples are washed twice to remove any residual dye, it is critical to use dyes that cannot be transferred between particles. Synaptosomes are well-labeled with calcein AM dyes (Gylys et al., 2000; Daniel et al., 2012; Prieto et al., 2017), lipophilic dyes that rapidly cross cell membranes and become trapped following hydrolysis of the AM ester. In addition to calcein AM and calcein red, we also used violet amine-reactive dye (covalent reaction with proteins) and MitoTracker Deep Red FM (thiol-conjugation and retention in mitochondria) to expand the number of dyes in some experiments. As shown in Extended Data Figure 3-1*A*, samples single-labeled with any of the four dyes do not exhibit any spectral overlap.

A representative set of density plots for a P2 sample single-labeled with calcein AM or calcein red, as well as a mixture of the two, on the Influx is shown in Figure 3*C*. Note that because even the background fluorescence is linearly related to FSC on the log-log plots, quadrant-based gating of double-positive events underestimates the number of positive events. To avoid this problem, we used polygon gating of each fluorophore individually versus FSC (Fig. 3*C*). Boolean logic is used to determine the number of double-positive events, which can be visualized by displaying only the positive-gated population of one fluorophore on a plot of FSC versus the other fluorophore (Fig. 3*C*, right).

Having verified the presence of calcein double-positive events in P2 mixtures (Fig. 3*C*), we sought experimental confirmation that the dyes did not transfer between particles. Because only the cleaved, polar dye molecules are retained, we reasoned that even if some membrane disruption and dye leak occurred during mixing and centrifugation, the polar molecules would be unable to label surrounding intact particles bearing the other calcein dye. We confirmed this by sonicating a P2 sample truly double-labeled with both calcein dyes and adding an unlabeled P2 sample to the sonicated solution using the exact same experimental workflow. As shown in Extended Data Figure 3-1*B*, no calcein AM-positive or calcein red-positive events were detected. This demonstrates that even after complete membrane disruption to liberate the entire dye content of one sample, we do not observe measurable transfer to an unlabeled sample after washing and mixing.

### Aggregation, but not coincidence, causes false double-positive events in P2 samples

**Figure 4.**
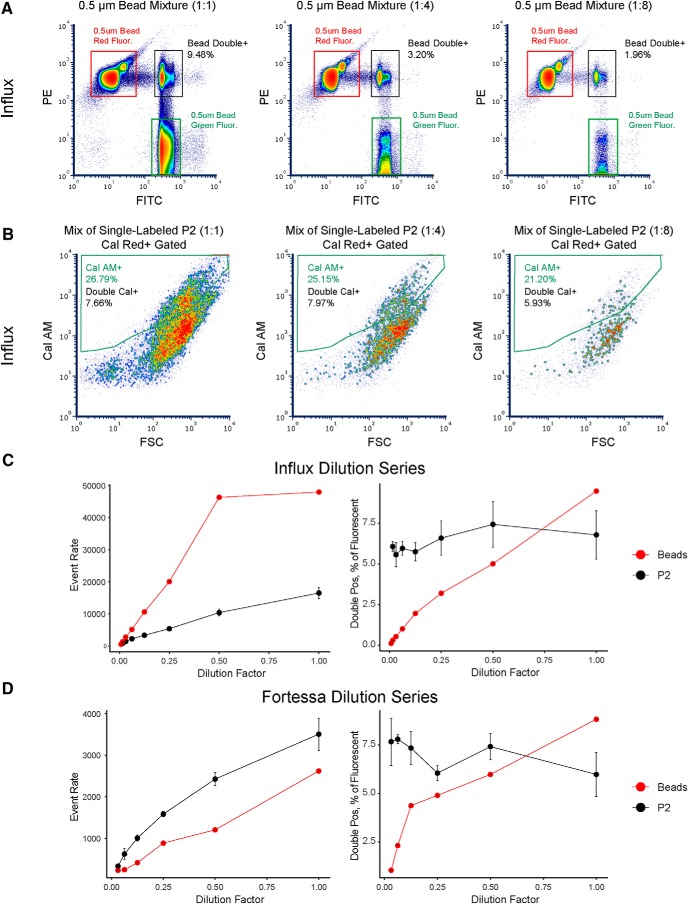
Dilution series reduces false double-positive bead events but not double-calcein positive P2 events. ***A***, Representative density plots of a dilution series for a mixture of fluorescent 0.5-µm PS beads detected using FSC triggering on the Influx. Individual PS beads and double-positive events are distinguished by their green (FITC detector) or red (PE detector) fluorescence. ***B***, Representative density plots of a dilution series for a mixture of single calcein-labeled P2 samples detected using FM triggering on the Influx. Only events gated positive for calcein red are displayed. ***C***, Measurements from full dilution series of bead mixture or single calcein-labeled P2 mixtures (*n* = 3) on the Influx. P2 data are displayed as mean ± SEM. Left, Event rate. Right, Double-positive events expressed as a percentage of all fluorescent events. ***D***, Measurements from full dilution series of bead or single calcein-labeled P2 mixtures on the Fortessa. Left, Event rate. Right, Double-positive events expressed as a percentage of all fluorescent events. See also Extended Data Figure 4-1.

With our multicolor fluorescence assay in hand, we turned toward distinguishing between coincidence and aggregation. Similar to previous studies (Libregts et al., 2018), we used mixtures of green- and red-fluorescent 500-nm PS beads to model coincidence. We repeated the dilution series experiments with mixtures of beads or mixtures of single calcein-labeled P2 samples on both Influx (Fig. 4*A*,*B*) and Fortessa (Extended Data Fig. 4-1*A*,*B*). For bead mixtures, we observed a clear population of double-positive events (red- and green-bead coincidences) on both instruments. At high concentrations, the Fortessa performed particularly poorly with regard to coincidence, with a wide smear of red-fluorescent bead multiplets and double-positive events (Extended Data Fig. 4-1*A*). On both instruments, we observed a linear reduction in the number of double-positive bead events with dilution, with virtually none detected at event rates <500 events/s (Fig. 4*C*,*D*). In contrast, the percentage of double-positive events from P2 samples remained relatively constant across the entire dilution series. P2 double-positive events persisted even at event rates <100 events/s, which strongly suggests that coincidence is not the source of these false double-positive events (Fig. 4*C*,*D*).

10.1523/ENEURO.0009-19.2019.f4-1 Extended Data Figure 4-1 Dilution series reduces false double-positive bead events but not double-calcein positive P2 events (Fortessa). ***A***, Representative density plots of a dilution series for a mixture of fluorescent 0.5-µm PS beads detected using FSC triggering on the Fortessa. PS beads and double-positive events are distinguished by their green (FITC detector) or red (PE detector) fluorescence. In addition to clear overlaps between green and red fluorescent beads, we also observed what appeared to be doublet, triplet, and higher order multiplets of the Nile Red 0.5-µm PS beads. We confirmed this based on the doubling and tripling of the red fluorescence intensity of the “double” and “triple” gated populations relative to the single gate. At high concentrations (left), these multiplets could also be detected together with the green fluorescent beads. ***B***, Representative density plots of a dilution series for a mixture of single calcein-labeled P2 samples detected using FM triggering on the Fortessa. Only events gated positive for calcein AM are displayed. Download Figure 4-1, TIF file.

**Figure 5.**
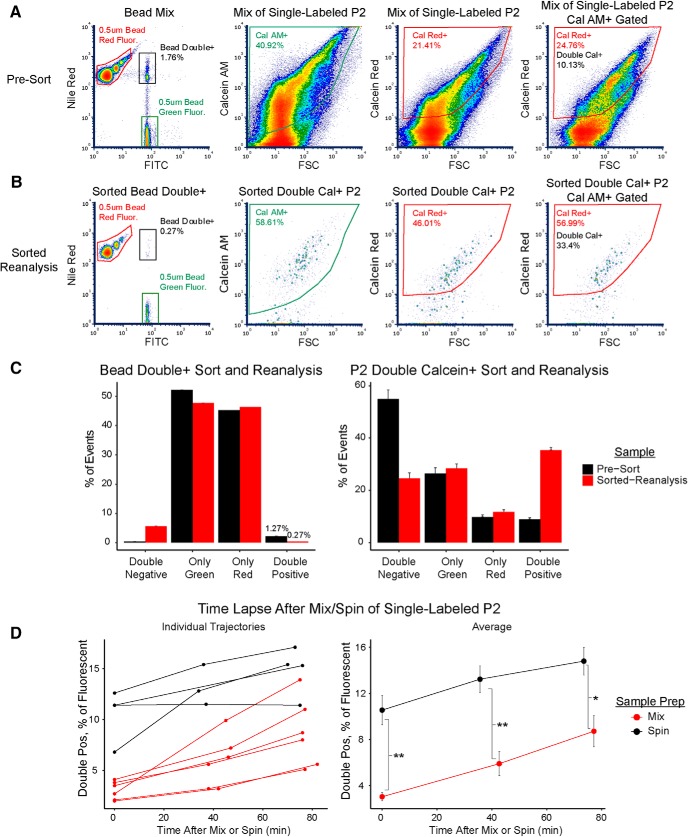
Effects of fluorescence-activated sorting, centrifugation resuspension, and time lapse on double-positive P2 events. These experiments were conducted using FM triggering on the Influx. ***A***, Representative density plots of pre-sorted samples. Left, Mixture of fluorescent 0.5-µm PS beads on the Influx. The “bead double+” gate was used for sorting. Mid-left and right, Mixture of single calcein-labeled P2 samples. Right, Calcein AM+ gated events from single calcein-labeled P2 samples were gated for calcein red+. This “double cal+” gate was used for sorting. ***B***, Representative density plots of sorted samples re-analyzed on the Influx. Left, Sample sorted for bead double+ gate above. Mid-left and right, Sample sorted for double cal+ gate above. Right, Calcein AM+ gated events from double cal+ sorted sample. ***C***, Summary of pre-sort versus reanalysis of sorted double-positive bead and P2 (*n* = 3) samples. P2 data are plotted as mean ± SEM. ***D***, Comparison of time lapse data for centrifugation-resuspension (“spin”) versus mixing without centrifugation (“mix”) of single-labeled P2 samples. Left, Time lapse of double-positive events plotted expressed as a percentage of all fluorescent events. Samples include the following mixtures of single-labeled P2 samples: violet amine-reactive dye/MitoTracker Deep Red FM (*n* = 2), calcein AM/calcein red (*n* = 3), violet amine-reactive dye/calcein AM (*n* = 1), violet amine-reactive dye/calcein AM/calcein red (*n* = 2), all four dyes (*n* = 2). Right, Comparison of averages for the individual trajectories shown on the left, plotted as mean ± SEM. A Student’s *t* test was performed comparing mix versus spin at each time point; *p < 0.05, **p < 0.01. See also Extended Data Figure 5-1.

We speculated that the double-positive events might come from the physical association of two separate fluorescent particles in the P2 samples. To further probe the nature of these events, we sorted double-positive events from bead or P2 mixtures on the Influx and reanalyzed the sorted material (Fig. 5*A*,*B*). Sorting of bead double-positive events failed to increase the frequency of these events in the sorted sample, with reanalysis showing a ∼4-fold decrease (Fig. 5*C*). Consistent with the dilution experiments, this result suggests that the majority of green- and red-fluorescent beads are not physically associated with each other during detection of double-positive events. In contrast, sorting of P2 double-positive events increased the frequency of these events ∼3-fold in the sorted sample reanalysis (Fig. 5*C*). We note that a 3-fold increase in fluorescent particles during sort reanalysis is similar to that achieved by (Biesemann et al., 2014) for fluorescent VGLUT1+ synaptosomes, suggesting that P2 double-positive events behave as stable, “single particles” in this experiment. It is also possible that the high pressures encountered during sorting physically disrupt some aggregates, as suggested by Biesemann (2010; his Results 3.2, Fig. 12, pp 79–81), which would further decrease the frequency of double-positive events in reanalysis.

10.1523/ENEURO.0009-19.2019.f5-1 Extended Data Figure 5-1 Scatter-based singlet gating strategies employed in conventional flow cytometry are not effective for P2 samples. A mixture of single calcein-labeled P2 samples was analyzed by FM triggering on the Influx or the Fortessa. Only events gated for either single calcein-positive events (top) or double calcein-positive events (bottom) are displayed. Left, The Influx is an analog cytometer and does not measure height and width of pulses for the FSC detector; we instead compared the width of the trigger pulse (FM4-64) to the FSC intensity (equivalent to “area” on digital cytometers such as the Fortessa). Right, Comparison of area and height of FSC pulses on the Fortessa. On both the Influx and Fortessa, both single-positive and double-positive events display a singlet linear profile. Download Figure 5-1, TIF file.

The divergent behavior of bead and P2 samples in dilution and sorting experiments strongly suggests that the majority of P2 double-positive events are aggregates. Since we routinely vortex samples at high speed for 5 s before flow cytometry, this suggests that the double-positive aggregates are relatively stable. We hypothesized that these aggregates form during centrifugation and are not fully disrupted during resuspension and filtration. To test this hypothesis, we conducted the multicolor dye-labeling assay with and without centrifuging the sample mixture and monitored the samples over time on the Influx. As shown in Figure 5*D*, centrifugation resuspension significantly increased the frequency of double-positive events compared to mixing without centrifugation. Intriguingly, the frequency of double-positive events increased over time for nearly every sample (Fig. 5*D*, left panel).

Taken together with the dilution and sorting experiments, these results suggest that particle aggregation in P2 samples is an ongoing process that is accelerated by centrifugation. We emphasize that our assay actually underestimates the frequency of true aggregates; green-green or red-red aggregates are not detected as double-positive events, but such aggregates are surely formed during centrifugation and washing of single-labeled samples. The mixing of single-labeled samples without centrifugation shown in Figure 5*D*, mix samples, actually underestimates the true proportion of aggregates, and we therefore conducted all other experiments in this study with centrifugation of the mixtures.

Aggregation of cells can also affect conventional flow cytometry experiments, and we wondered whether commonly employed “singlet gating” procedures could discriminate aggregates in our P2 samples. We plotted the trigger pulse width (in this case, FM4-64) versus FSC on the Influx, or FSC-H versus FSC-A on the Fortessa, for events gated as single or double calcein-positive (Extended Data Fig. 5-1). Cell doublets generally deviate from linearity on such plots, forming a “cloud” that can be gated apart from the linear singlet profile (Wersto et al., 2001). We found no such deviation from linearity in single-positive gated events, which represent the majority of the sample. Although they bear only one fluorophore, single-positive events are expected to contain at least as many or even more aggregates than double-positive gated events (i.e., green-green, red-red, red-nonfluorescent, green-nonfluorescent, etc.). The lack of deviation from linearity for any events in these pulse profiles suggests that a “doublet profile” does not exist for our P2 samples. Indeed, a recent flow cytometry study using this gating procedure in analysis of FSC-triggered P2 samples found that all their events obeyed this “linear singlet profile” (Prieto et al., 2017). The lack of a distinguishing doublet profile becomes especially apparent when looking only at double-positive events, which are predominantly aggregates and display a similar linear pulse profile to single-positive events (Extended Data Fig. 5-1). Thus, we found no evidence that pulse profile is an effective means of removing false double-positive aggregates from downstream analysis of submicron particles.

### Double-positive event frequency increases with FSC and is reduced in nonionic buffer

**Figure 6.**
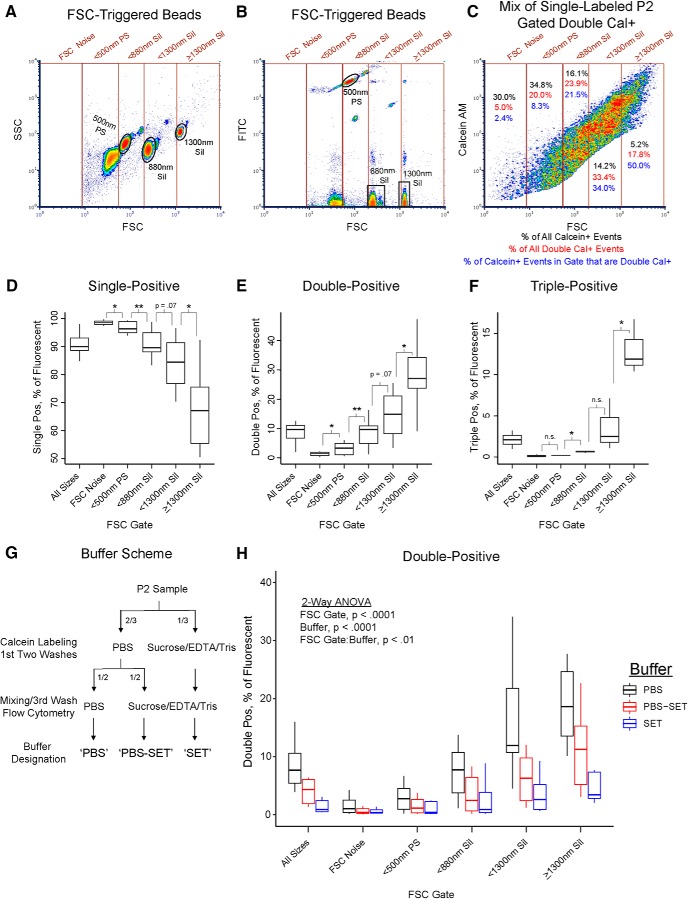
Distribution of double-positive P2 events across FSC ranges and in different sample buffers. These experiments were conducted using FM triggering on the Influx. ***A***, Representative density plot of non-fluorescent silica and fluorescent PS beads (Apogee) showing the five FSC-gated regions based on noise threshold and bead positions. ***B***, Same sample and FSC gates as in ***A*** but plotted to display green fluorescence of PS beads (FITC detector). ***C***, Representative density plot showing the five FSC-gated regions on a mixture of single calcein-labeled P2 samples. Only gated double-positive events are displayed. The percentage of all fluorescent (i.e., calcein-labeled) events that falls into each FSC gate is displayed in black (all five FSC gates sum to 100%). The percentage of all calcein double-positive events that falls into each FSC gate is displayed in red (all five FSC gates sum to 100%). The number of double-positive events within each FSC gate, expressed as a percentage of all fluorescent events in that FSC gate, is displayed in blue. ***D–F***, Box and whiskers plots of single-positive, double-positive, or triple-positive events, expressed as a percentage of all fluorescent events within the respective FSC gate. Central bar represents the median. Lower and upper edges correspond to 25th and 75th percentiles. Lower and upper whiskers extend to the smallest or largest value no greater than 1.5 times the interquartile range away from the corresponding edge. Data for single-positive and double-positive events are derived from two-color mixtures of P2 samples single-labeled with calcein AM/calcein red (*n* = 4), violet amine-reactive dye/MitoTracker Deep Red FM (*n* = 1) or calcein AM (*n* = 1) or calcein red (*n* = 1), as well as three-color mixtures single-labeled with violet amine-reactive/calcein AM/calcein red (*n* = 3). Triple-positive event data are derived only from the three-color mixtures. A Student’s *t* test was performed comparing each FSC gate to that directly above or below it; *p < 0.05, **p < 0.01. ***G***, Diagram of buffer scheme during sample preparation. PBS and SET samples are labeled, washed, and run on the flow cytometer in their respective buffers. PBS-SET samples undergo calcein labeling and the first two washes in PBS before mixing, a third wash, and flow cytometry in SET buffer. ***H***, Box and whiskers plot of double-positive events, expressed as a percentage of all fluorescent events within the respective FSC gate. Data are derived from two-color mixtures of P2 samples single-labeled with calcein AM/calcein red using the indicated buffer scheme: PBS (*n* = 10), PBS-SET (*n* = 8), or SET (*n* = 10). Two-way ANOVA was performed, revealing significant main effects of FSC gate (Df = 5, *F* = 20.06, *p* = 2.7e-15) and buffer (Df = 2, *F* = 20.14, *p* = 1.8e-8), as well as a significant interaction (Df = 10, *F* = 2.78, *p* = 0.004). See also Extended Data Figure 6-1.

Although bead standards do not provide accurate size estimation of biological particles, we wondered whether the relative size information afforded by FSC intensity would correlate with the presumably larger size of aggregates. We analyzed five discrete regions ranging from low to high FSC on the Influx, based on beads and the FSC noise threshold (Fig. 6*A*,*B*). A representative overlay of the FSC gates onto gated double-positive P2 events is shown in Figure 6*C*. Strikingly, the top two FSC gates contain ∼50% of all double-positive events (percentages in red) but only ∼19% of all calcein-positive events (percentages in black), while the bottom two FSC gates contain only ∼25% of all double-positive events among ∼65% of all calcein-positive events. Accordingly, the percentage of fluorescent events within each FSC gate that were double-positive steadily increased with FSC (Fig. 6*C*, percentages in blue). For all fluorescent events within each FSC gate, we quantified the percentage of single-, double-, and triple-positive events on the Influx (Fig. 6*D–F*). Although the overall frequency of single-positives was ∼90%, this steadily decreased from ∼97% in the FSC Noise region to ∼70% in the region above or equal to 1300-nm silica beads (Fig. 6*D*). The overall frequency of double-positives was ∼10%, but steadily increased from ∼2% in the FSC Noise region to ∼27% in the region above or equal to 1300-nm silica beads (Fig. 6*E*). Similarly, the overall frequency of triple-positives was ∼2%, but increased from ∼0% in the FSC noise range to ∼12% in the region above or equal to 1300-nm silica beads (Fig. 6*F*). These results suggest that although aggregates are present in all FSC regions, their presence is strongly correlated with increasing FSC. Again, we emphasize that our fluorescence assay underestimates aggregation, even in samples where the measured double-positive frequency exceeds 40% in the highest FSC regions (Fig. 6*C*).

10.1523/ENEURO.0009-19.2019.f6-1 Extended Data Figure 6-1 Effects of different sample buffers on calcein labeling, FSC intensity, and fluorescence measurements. These experiments were conducted on the Influx using FSC triggering (for beads in ***B***) or FM triggering (for P2 samples in ***A***, ***C***). ***A***, Representative density plots for mixtures of single calcein-labeled P2 samples. Columns indicate the buffer designation for each sample, while the rows indicated the gated population displayed. ***B***, FSC and green fluorescence (FITC detector) of fluorescent 0.5- and 0.8-µm PS beads (Bangs Labs; B) run in PBS (left) or SET (right) buffer. The median and SD of FSC and green fluorescence signal intensity are shown below for both gated bead populations. ***C***, Box and whiskers plots of fluorescent events (all events positive for either calcein), expressed as a percentage of all events. Central bar represents the median. Lower and upper edges correspond to 25th and 75th percentiles. Lower and upper whiskers extend to the smallest or largest value no greater than 1.5 times the interquartile range away from the corresponding edge. Data are derived from the same single calcein-labeled P2 mixtures presented in Figure 6*H* (*n* = 10 for PBS, *n* = 8 for PBS-SET, and *n* = 10 for SET). A Student’s *t* test was performed comparing each buffer pair; *p < 0.05. Download Figure 6-1, TIF file.

Several groups familiar with microscopic analysis of synaptosomal preparations have noted that ionic buffers (e.g., PBS) cause synaptosomes to aggregate, while nonionic media (e.g., sucrose buffer) favors an even distribution of single particles (Choi et al., 2009; Daniel et al., 2012). We repeated our double calcein assay on the Influx using the SET buffer recommended by Daniel et al. (2012). Because many assays are not possible in nonionic media, we tested labeling in PBS and then switching to SET for flow cytometry (designated PBS-SET) in addition to conducting the entire procedure in SET (Fig. 6*G*). Compared to PBS, both PBS-SET and SET samples displayed similar FSC versus FM4-64 profiles (Extended Data Fig. 6-1*A*), indicating that SET did not impair FM-triggered particle detection or FSC measurement. FSC and green-fluorescence measurements of PS beads in PBS and SET were also indistinguishable (Extended Data Fig. 6-1*B*). However, we noticed that the total number of calcein-positive events was reduced to ∼20% in when calcein labeling was conducted in SET, compared to ∼40% for PBS or PBS-SET (Extended Data Fig. 6-1*C*). We suspect that the nonionic media and divalent cation sequestration by EDTA somewhat reduce the esterase activity of synaptosomes; nonetheless, the labeling efficiency was sufficient to analyze a large number of labeled particles present in these mixtures. As shown in Figure 6*H*, the percentage of fluorescent events within each FSC gate that were double-positive was reduced in SET compared to PBS. A stepwise pattern was observed, wherein the double-positive frequency was highest for PBS, lower for PBS-SET, and lowest for SET. Two-way ANOVA revealed highly significant main effects of both FSC gate (Df = 5, *F* = 20.06, *p* = 2.7e-15) and buffer (Df = 2, *F* = 20.14, *p* = 1.8e-8) as well as a significant interaction (Df = 10, *F* = 2.78, *p* = 0.004). The significant interaction makes sense given that buffer has little effect in the lower FSC regions, while the reduction of double-positive events is greater in the higher FSC regions (Fig. 6*H*). These results provide further evidence that double-positive events are aggregates, and that such aggregates are less abundant in nonionic SET buffer.

### False colocalization of antigens in immunostained P2 samples

**Figure 7.**
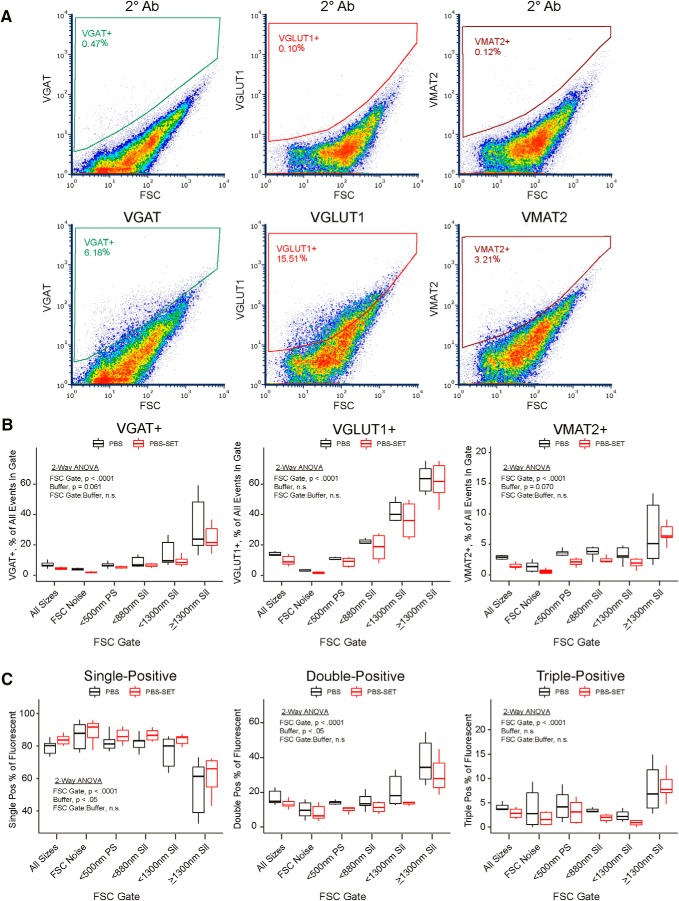
Fractional abundance of immunostained P2 events in different FSC ranges and in different sample buffers. These experiments were conducted using FM triggering on the Influx. ***A***, Representative density plots of P2 samples immunostained for VGAT, VGLUT1, and VMAT2 and run in PBS. Top, Secondary-only controls in which primary antibodies were omitted. Bottom, Immunostained with indicated primary antibodies. ***B***, Box and whiskers plot of immunostained events as indicated, expressed as a percentage of all events within the respective FSC gate. Central bar represents the median. Lower and upper edges correspond to 25th and 75th percentiles. Lower and upper whiskers extend to the smallest or largest value no greater than 1.5 times the interquartile range away from the corresponding edge. Data are derived from P2 samples triple stained and washed in PBS, and subsequently run in either PBS (*n* = 6) or SET (*n* = 6) as sample buffer. Two-way ANOVA was performed for each dataset. Left, For VGAT+ events, FSC gate (Df = 5, *F* = 21.51, *p* = 2.9e-12) and buffer (Df = 1, *F* = 4.70, *p* = 0.034) were both significant, with no significant interaction (Df = 5, *F* = 0.40, *p* = 0.85). Middle, For VGLUT1+ events, only FSC gate (Df = 5, *F* = 68.9, *p* < 2e-16) was significant, while buffer (Df = 1, *F* = 0.36, *p* = 0.55) and the interaction (Df = 5, *F* = 0.19, *p* = 0.97) were not. Right, For VMAT2+ events, FSC gate (Df = 5, *F* = 12.54, *p* = 2.4e-8) and buffer (Df = 1, *F* = 5.21, *p* = 0.026) were both significant, with no significant interaction (Df = 5, *F* = 0.27, *p* = 0.93). ***C***, Box and whiskers plots of single-positive, double-positive, and triple-positive events expressed as a percentage of all fluorescent events within the respective FSC gate. Data are derived from the same immunostained P2 samples as in ***B***. Two-way ANOVA was performed for each dataset. Left, For single-positive events, FSC gate (Df = 5, *F* = 19.83, *p* = 1.3e-11) and buffer (Df = 1, *F* = 6.74, *p* = 0.012) were both significant, with no significant interaction (Df = 5, *F* = 0.15, *p* = 0.98). Middle, For double-positive events, FSC gate (Df = 5, *F* = 23.25, *p* = 6.6e-13) and buffer (Df = 1, *F* = 8.00, *p* = 0.006) were both significant, with no significant interaction (Df = 5, *F* = 0.40, *p* = 0.85). Right, For triple-positive events, only FSC gate (Df = 5, *F* = 8.37, *p* = 4.7e-6) was significant, while buffer (Df = 1, *F* = 1.91, *p* = 0.17) and the interaction (Df = 5, *F* = 0.19, *p* = 0.97) were not. See also Extended Data Figures 7-1, 7-2, 7-3.

Given reports claiming high purity of synaptosomes in FSC ranges between 500- to 1500-nm PS beads (Gylys et al., 2004; Postupna et al., 2014; Prieto et al., 2017), we suspected that many of the aggregates in our P2 samples contain synaptosomes. Although the violet amine reactive and calcein dyes efficiently label many particles in P2 samples, they do not provide information about the identity of these particles. We conducted three-color immunostaining of P2 samples on the Influx, targeting presynaptic markers expressed by excitatory, inhibitory, and monoaminergic neurons, respectively: VGLUT1, VGAT, and vesicular monoamine transporter 2 (VMAT2). Although we did not attempt immunostaining in SET buffer, we did mimic the PBS-SET condition described above by resuspending stained and washed samples in SET for flow cytometry. As shown in Figure 7*A*, immunopositive events for all three transporters were observed across the entire FSC range and reflect their abundance in the brain (VGLUT1 > VGAT > VMAT2). Looking at all immunopositive events across FSC gates (Extended Data Fig. 7-1*A*), VGAT+ and VMAT2+ events displayed an asymmetric distribution centered below 500-nm PS beads, while VGLUT1+ events were distributed fairly uniformly just below 880-nm silica beads. Although the distribution of all immunopositive events across FSC gates was not affected by buffer (Extended Data Fig. 7-1*B*), the percentage of immunopositive events within each FSC gate was slightly reduced in PBS-SET samples (Fig. 7*B*). Two-way ANOVA revealed a significant main effect of buffer for VGAT (Df = 1, *F* = 4.70, *p* = 0.034) and VMAT2 (Df = 1, *F* = 5.21, *p* = 0.026) but not VGLUT1 (Df = 1, *F* = 0.36, *p* = 0.55). A highly significant main effect of FSC gate was observed for all three markers (Df = 5, *F* = 21.51, *p* = 2.9e-12 for VGAT; Df = 5, *F* = 68.9, *p* < 2e-16 for VGLUT1; Df = 5, *F* = 12.54, *p* = 2.4e-8 for VMAT2), where the percentage of immunopositive events within each FSC gate increased with FSC (Fig. 7*B*). All buffer × FSC gate interactions were non-significant. Consistent with recent studies (Prieto et al., 2017), the percentage of VGLUT1+ events in the highest FSC gate approaches ∼60%.

10.1523/ENEURO.0009-19.2019.f7-1 Extended Data Figure 7-1 Distribution of immunostained P2 events across FSC ranges and comparison between single-positive and double-positive event frequencies. All data are derived from the same samples as presented in Figure 7, i.e., P2 samples immunostained in PBS for VGAT, VGLUT1, and VMAT2 and run on the Influx with FM triggering in PBS or SET as sample buffer. ***A***, Representative histogram with overlay of FSC gates. Only events gated as positive for VGAT (left), VGLUT1 (middle), or VMAT2 (right) are displayed. ***B***, Box and whiskers plots of immunopositive events within each FSC gate, expressed as a percentage of all immunopositive events for the indicated antigen. Analogous to the histograms above, the sum across FSC gates is 100% for any one sample. Central bar represents the median. Lower and upper edges correspond to 25th and 75th percentiles. Lower and upper whiskers extend to the smallest or largest value no greater than 1.5 times the interquartile range away from the corresponding edge. ***C***, Relative abundance of all events immunopositive for a given antigen, expressed as a percentage of all events. Data for are plotted as mean ± SEM for PBS or PBS-SET (*n* = 6 for each). ***D***, left, A simple model for collisions of single-positive events leading to double-positive events is given by sampling with replacement. The relative abundance of any given double-positive event is estimated by multiplying the relative abundances of the two single-positive events. Right, Comparison of the observed and expected frequency for each type of double-positive event, expressed as a percentage of all double-positive events. Observed data represent the average for PBS or PBS-SET (*n* = 6 for each). ***E***, Alexa Fluor 488 MESF calibration bead standards (left) used to construct a calibration curve based on measured median fluorescence intensity and the manufacturer’s stated MESF for each bead (right). ***F***, A P2 sample stained for VGAT (with anti-mouse Alexa Fluor 488 secondary) was gated for immunonegative and immunopositive events as shown in Figure 7*A*. Plotted are the median fluorescence intensity and rSD for each FSC gate within the immunostaining gate. Download Figure 7-1, TIF file.

10.1523/ENEURO.0009-19.2019.f7-2 Extended Data Figure 7-2 Single-, Double-, and Triple-Positive Percentages for Each Type of Immunopositive Event. Data derived from samples shown in Fig. 7B-C (Mean ± SEM for n=6 replicates each of buffer). Download Figure 7-2, DOC file.

10.1523/ENEURO.0009-19.2019.f7-3 Extended Data Figure 7-3 MESF Calibration of AlexaFluor488 Immunostaining. Numerical data corresponding to those shown in Figure 7-1E-F. Download Figure 7-3, DOC file.

Although specific cases of neurotransmitter co-transmission and co-release have been observed (for review, see Hnasko and Edwards, 2012; Vaaga et al., 2014), the expression of VGLUT1, VGAT, and VMAT2 should be mutually exclusive for the majority of presynaptic terminals. For all immunopositive events within each FSC gate, we quantified the percentage of single-, double-, and triple-positive events (Fig. 7*C*). Similar to double calcein-positive events, we observed a steady decrease in single-positive and a steady increase in double-positive and triple-positive events with increasing FSC. Two-way ANOVA revealed a significant main effect of FSC gate for each event type (Df = 5, *F* = 19.83, *p* = 1.3e-11 for single-positive; Df = 5, *F* = 23.25, *p* = 6.6e-13 for double-positive; Df = 5, *F* = 8.37, *p* = 4.7e-6 for triple-positive). PBS-SET samples had higher single-positive event frequencies and lower double-positive event frequencies compared to PBS (Fig. 7*C*). The main effect of buffer was significant for single-positive (Df = 1, *F* = 6.74, *p* = 0.012) and double-positive (Df = 1, *F* = 8.00, *p* = 0.006), but not for triple-positive events (Df = 1, *F* = 1.91, *p* = 0.17). None of the buffer × FSC gate interactions were significant. Thus, although PBS-SET samples did display reduced aggregation, the effect was modest, especially for triple-positive events. Similar to the percentage of immunopositive events within each FSC gate, the frequency of false co-localization increases with FSC. To further illustrate this point, we calculated the percentage of immunopositive events for each antigen that were single-, double-, or triple-positive within each FSC gate (Extended Data Fig. 7-2). In the FSC noise region we found that ∼80% of VGAT+, ∼75% of VGLUT1+, and ∼63% of VMAT2+ events were single-positive. Strikingly, in the highest FSC gate, the single-positive percentage was only ∼12% for VGAT+, ∼51% for VGLUT1+, and ∼9% for VMAT2. These results suggest that many of the putative “synaptosomal” events in the upper FSC regions are aggregates containing multiple synaptosomes.

The quantification of double-positive events described above represents the sum of the three possible subtypes (i.e., VGAT+/VGLUT1+, VGLUT1+/VMAT2+, and VMAT2+/VGAT+). We wondered whether the frequency of these double-positive subtypes was related to the overall abundance of their respective antigens (Extended Data Fig. 7-1*C*). We used the percentage of all events immunopositive for each antigen to construct a simple probability model. In this model, a double-positive event represents the random sampling of two events with replacement. The probability of a particular double-positive subtype is therefore obtained by multiplying the frequencies of its respective event types (Extended Data Fig. 7-1*D*). Consistent with the expected frequencies of double-positive subtypes, we found that VGLUT1+/VGAT+ events were by far the most abundant (∼75%), followed by VGLUT1+/VMAT2+ (∼20%), and finally VMAT2+/VGAT+ (∼5%; Extended Data Fig. 7-1*D*). These results are consistent with a simple “collision” model of aggregation in the P2 crude synaptosome preparation. Furthermore, they strongly suggest that many double-positive events observed in these immunostaining experiments are aggregated particles rather than single synaptosomes co-expressing multiple vesicular transporters.

Although scatter measurements of submicron particles may vary dramatically across cytometers, fluorescence measurements can be readily calibrated. We determined the fluorescence intensity of VGAT/anti-mouse Alexa Fluor 488 immunostaining in molecules of equivalent soluble fluorophore (MESF) using calibrated bead standards (Extended Data Fig. 7-1*E*). Because particle fluorescence is directly related to FSC intensity regardless of the immunostaining procedure, many immunopositive events at a given FSC intensity would exhibit fluorescence intensity less than or equal to immunonegative events at a higher FSC intensity nearby. We therefore analyzed the median fluorescence intensity of gated immunopositive and immunonegative events within each FSC gate (Extended Data Fig. 7-1*F*). Numerical MESF data are also included in Extended Data Figure 7-3, which should facilitate comparison across laboratories.

### Particle recovery following repeated centrifugation

**Figure 8.**
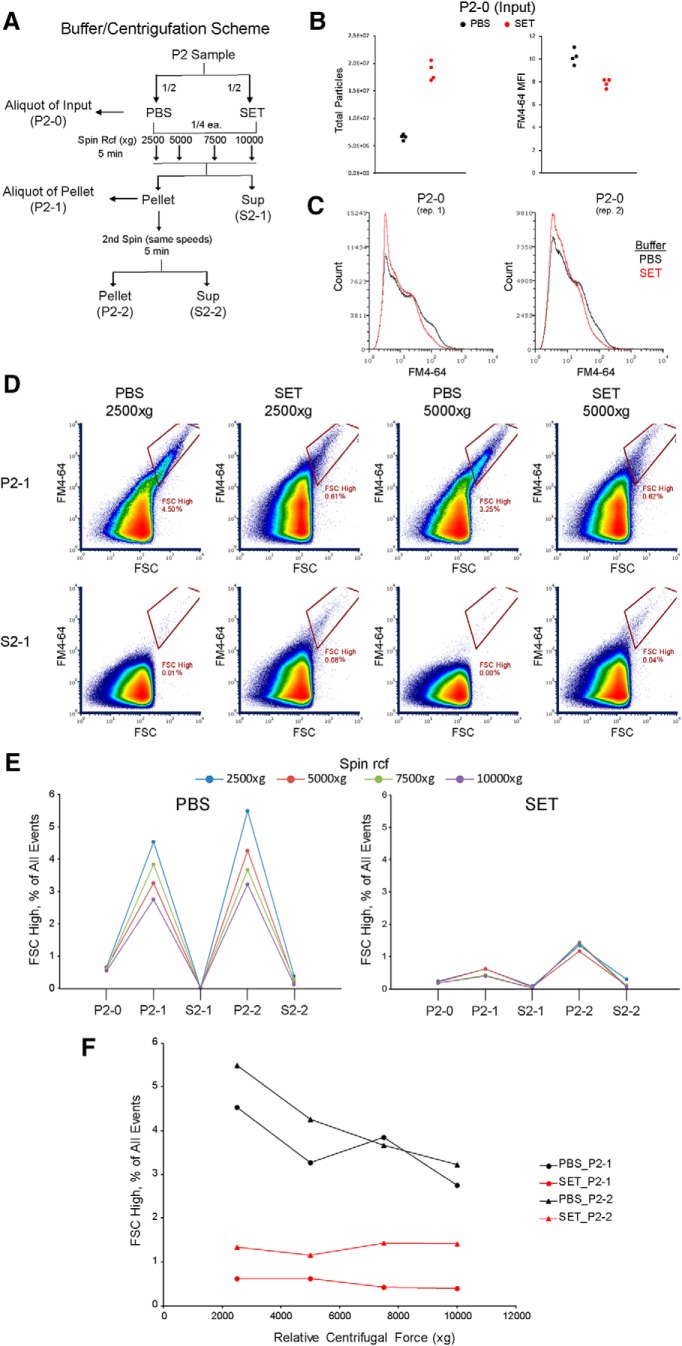
Effects of repeated centrifugation in different sample buffer on particle abundance and composition of P2 samples. These experiments were conducted using FM triggering on the Influx. ***A***, Diagram of buffer/centrifugation scheme. A P2 sample resuspended in PBS or SET was aliquoted to measure input (P2-0), followed by two centrifugations for 5 min at the indicated relative centrifugal force (× *g*). An aliquot of the first pellet (P2-1) is reserved before the second spin. All samples are then analyzed by flow cytometry using absolute counting beads to measure particle number in each fraction. ***B***, Input samples (P2-0) in PBS or SET buffer (*n* = 4 each). Left, Total particle counts. Right, Median fluorescence intensity of FM4-64 for all events. ***C***, Representative histogram of FM4-64 fluorescence of all events for two replicate input samples (P2-0) in PBS and SET buffer. ***D***, FSC versus FM4-64 density plots for P2-1 (top) or S2-1 (bottom) samples in PBS or SET buffer centrifuged at 2500 × *g* (left) or 5000 × *g* (right). FSC high gate corresponds to the top two FSC gates from Figures 6, 7. ***E***, Gated FSC high events, expressed as % of all events, plotted for PBS (left) or SET (right) in each of five fractions moving through the centrifugation protocol at indicated relative centrifugal force (× *g*). Left to right in each panel, Input (P2-0), first pellet (P2-1) and supernatant (S2-1), second pellet (P2-2) and supernatant (S2-2). ***F***, Gated FSC high events, expressed as % of all events, for P2-1 and P2-2 samples in PBS or SET at indicated relative centrifugal force (× *g*). See also Extended Data Figure 8-1.

Given the increased frequency of aggregates after centrifugation (Fig. 5*D*), in higher FSC regions (Fig. 6*D–F*), and in PBS (Fig. 6*H*), we wondered whether buffer and centrifugation might interact to bias sample composition toward aggregates following repeated centrifugations. Indeed, Gray and Whittaker (1962) previously reported increased recovery of acetylcholine in the low-speed P1 pellet when using saline instead of sucrose buffer, which was interpreted as aggregation in saline increasing the effective size of synaptosomes (and thus sedimentation at lower speed). To assess whether multiple centrifugation steps might bias particle recovery in PBS versus SET, we conducted two sequential 5-min centrifugations of P2 samples in PBS or SET buffer at varying speeds and used flow cytometry with absolute counting beads to determine the particle concentration of the pellets and supernatants (Fig. 8*A*). We found that under carefully controlled conditions with the absolute counting beads, PBS reduced particle abundance by ∼3-fold before any centrifugation (Fig. 8*B*). It is possible that certain particles in the P2 sample are susceptible to destruction in PBS, but several features of the data suggest that aggregation contributes to the reduction in particle counts. First, the median fluorescence intensity of membrane staining by FM4-64 was higher in PBS (Fig. 8*B*), which was due to increased relative abundance of brighter, larger events with higher FSC (Fig. 8*C*,*D*). In line with the findings of Gray and Whittaker (1962), we found that these “FSC high” particles were more efficiently pelleted in PBS at lower relative centrifugal force (2500–5000 × *g*) compared to SET buffer (Fig. 8*D*). The relative abundance of these particles was dramatically increased in the two pellets, P2-1 and P2-2, compared to the input sample and two supernatants (Fig. 8*E*). The relative abundance of the large particles in PBS samples was highest in 2500 × *g* pellets and lowest in 10,000 × *g* pellets, while no clear relationship between relative abundance and centrifugation speed was observed in SET buffer (Fig. 8*F*). A slight increase in these FSC high particles was observed when comparing spin 1 versus spin 2 for both buffers, but this effect was modest compared to the increase in PBS versus SET (Fig. 8*F*).

10.1523/ENEURO.0009-19.2019.f8-1 Extended Data Figure 8-1 Particle recovery in P2 samples following repeated centrifugations. All data are derived from the samples presented in Figure 8, i.e., P2 samples subjected to two centrifugations for 5 min in PBS or SET buffer at varying relative centrifugal force (× *g*). Absolute counting beads were added to each sample to determine absolute particle counts. ***A***, Total particle counts in each fraction for P2-1, S2-1, P2-2, and S2-2 samples in PBS. ***B***, Same as ***A*** but for SET buffer. ***C***, Percent recovery in the pellet following each centrifugation at indicated rcf (× *g*). ***D***, Total particle counts for pellets and supernatants across varying centrifugation rcf (× *g*) and in PBS or SET buffer for spin 1. ***E***, Same as ***D*** but for spin 2. Download Figure 8-1, TIF file.

As expected, the overall efficiency of particle recovery in the pellet of each spin increased with centrifugation speed, with the exception of the first spin in PBS (Extended Data Fig. 8-1*A–C*). Similar to the input samples, it appears that a combination of aggregation and/or destruction contribute to the loss of particle counts in the first PBS spin, although the recovery in the second spin was higher than in SET buffer. Critically, the first 5-min spin at 2500–5000 × *g* in either buffer leaves over half the P2 particles in the supernatant (Extended Data Fig. 8-1*D*). Collectively, these data suggest that P2 samples do undergo a certain degree of further fractionation during repeated centrifugation-based wash steps. Although some centrifugation bias is inevitable regardless of buffer and centrifugation speed, our data suggest that lower centrifugation speeds and ionic buffers will bias the overall sample composition toward larger particles that are more likely to be aggregates.

## Discussion

The goal of most flow cytometry experiments is to accurately detect and quantify the fluorescence intensity of single particles or cells in suspension. Toward that goal, we employed a variety of experimental assays that can be used to optimize synaptosome flow cytometry experiments. We have summarized our approach in Table 5 and will mirror the workflow in our discussion. We note that this workflow could be employed for any type of small particle, but our discussion will focus on aspects specific to synaptosomes.

**Table 5.** Summary of key steps in microparticle flow cytometry experimental workflow

Purpose	Relevant figures	Experimental assays	Key notes
Determine detection sensitivity of cytometer	Fig. 1; Extended Data Fig. 1-1	FSC vs fluorescence triggering with lipid dye (e.g., FM4-64 or other) PS and silica bead standards	A number of dyes are suitable for fluorescence triggering (Gray et al., 2015; Arraud et al., 2016; Kormelink et al., 2016; Stoner et al., 2016). Multiple studies, including ours, have demonstrated the increased sensitivity, accuracy, and reliability of fluorescence triggering (Nolan and Stoner, 2013; Arraud et al., 2016). FSC signal from bead standards (especially PS) does NOT accurately reflect the size of biological particles detected. Refractive index differences lead to size underestimation, the extent of which is highly cytometer dependent (Table 6).
Determine linear range of particle detection	Fig. 2; Extended Data Fig. 2-1	Dilution series	Conducting a dilution series is critical to identify the range of linear detection on each cytometer.
Detection of coincidence and aggregation	Fig. 3; Extended Data Fig. 3-1	Multicolor dye labeling	A number of dyes are suitable for multicolor dye labeling assays, as described in cellular “barcoding” assays (Krutzik and Nolan, 2006; Krutzik et al., 2011).
Distinguishing between coincidence and aggregation	Fig. 4; Extended Data Fig. 4-1 Fig. 5	(1) Dilution series with multicolor dye labeling (2) Sorting double-positive events from beads vs samples (3) Spin vs mix multicolor dye labeling	We found that mixtures of different color PS beads are a suitable model of coincidence. Double-positive sample events that do not decrease with dilution, and those that can be enriched by sorting, strongly suggest aggregates vs coincidence. This can be further supported by testing whether the frequency of double-positive events is increased by centrifugation.
Reduce aggregation	Fig. 6; Extended Data Fig. 6-1 Fig. 7; Extended Data Fig. 7-1	Use of nonionic buffers	As reported in previous microscopy studies (Choi et al., 2009; Daniel et al., 2012), we found that nonionic sample buffers tend to reduce aggregation of synaptosomes. This is not a complete solution to eliminating false double-positive and triple-positive events.
Identify suitable FSC ranges	Fig. 6; Extended Data Fig. 6-1 Fig. 7; Extended Data Fig. 7-1	Immunostaining and multicolor dye labeling	FSC of beads provides relative references for avoiding regions with high false double-positive rates
Assess false colocalization of antigens	Fig. 7; Extended Data Fig. 7-1	Immunostaining of mutually exclusive antigens	Highly abundant antigens that should be mostly exclusive are best for these experiments.

### FSC triggering and bead-based size estimation

It is now widely accepted that FSC-based size estimation using PS beads underestimates biological particle size (Nolan, 2015; Lannigan et al., 2016). This consequence of the refractive index mismatch has been experimentally validated in numerous flow cytometry studies (Chandler et al., 2011; van der Pol et al., 2012; Simonsen, 2016). The extent of underestimation depends on a variety of factors including the scattering parameters used, refractive indices of the sample and beads, and optical configuration of the cytometer. Estimates of the lipid vesicle size range defined by PS beads have been conducted on various cytometers and are shown in Table 6.

**Table 6.** Previous comparisons of PS bead versus lipid vesicle scattering measurements

Study	Cytometer model	Parameter	PS bead range	Vesicle range
Chandler et al. (2011)	Apogee A40	FSC	0.5–1.0 µm	1.4–2.7 µm
van der Pol et al. (2012)	Beckman Coulter FC 500	FSC	0.5–0.9 µm	1.00–1.75 µm
van der Pol et al. (2012)	Apogee A40	FSC	0.5–0.9 µm	1.25–2.0 µm
van der Pol et al. (2012)	Becton Dickinson FACSCalibur	SSC	0.5–0.9 µm	2.3–4.6 µm
Simonsen (2016)	Becton Dickinson LSRII	SSC	110 nm	400 nm

Synaptosomes are heterogeneous in size, but a generally accepted size range is 0.5–1.0 µm (Gray and Whittaker, 1962; Hollingsworth et al., 1985; Dunkley et al., 1986, 1988; Williams et al., 2009; Fernández-Busnadiego et al., 2013). Although certain preparations contain larger synaptosomes, such as Dunkley’s Percoll fraction 4 from striatum (Robinson et al., 1989), most studies of cortical synaptosomes consistently obtain mean diameters of ∼500–600 nm (Table 7). Based on the synaptosome size ranges in Table 7 and vesicle size ranges defined by PS beads in Table 6, a majority of single synaptosomes would be expected to produce lower FSC signals than 500-nm PS beads and therefore would be undetectable by FSC triggering on many cytometers. Indeed, we could detect neither 585-nm silica beads nor more than half of P2 samples by FSC triggering on the Fortessa (Fig. 1*C*,*D*). Although the greater sensitivity afforded by the Influx enabled detection of 585-nm silica beads above the FSC noise threshold, more than half of the P2 sample was still undetectable by FSC triggering (Fig. 1*C*,*D*). Our results are consistent with those of Biesemann et al. (2014), who sorted fluorescent VGLUT1 synaptosomes from below the FSC noise threshold using FM triggering on a BD FACS Aria. These electron microscopy, immunostaining, and proteomic studies convincingly demonstrated that single synaptosomes can be purified by sorting events that fall below the FSC noise threshold, which was well below 750-nm PS beads on their cytometer (Biesemann, 2010; his Results 3.5, Fig. 15, pp 87–90).

**Table 7.** Previous size range measurements of mammalian synaptosomes

Study	Method	Species/region	Synaptosome fraction	Mean diameter (±SEM)
Dunkley et al. (1986)	Electron microscopy	Rat/cortex	P2 Percoll fraction 3	515 ± 17 nm
Dunkley et al. (1986)	Electron microscopy	Rat/cortex	P2 Percoll fraction 4	568 ± 14 nm
Dunkley et al. (1986)	Electron microscopy	Rat/cortex	P2 Percoll fraction 5	539 ± 12 nm
Dunkley et al. (1988)	Electron microscopy	Rat/cortex	S1 Percoll fraction 1	320 ± 130 nm
Dunkley et al. (1988)	Electron microscopy	Rat/cortex	S1 Percoll fraction 2	460 ± 150 nm
Dunkley et al. (1988)	Electron microscopy	Rat/cortex	S1 Percoll fraction 3	550 ± 130 nm
Dunkley et al. (1988)	Electron microscopy	Rat/cortex	S1 Percoll fraction 4	640 ± 120 nm
Dunkley et al. (1988)	Electron microscopy	Rat/cortex	S1 Percoll fraction 5	630 ± 190 nm
Robinson et al. (1989)	Electron microscopy	Rat/striatum	S1 Percoll fraction 3	591 ± 9 nm
Robinson et al. (1989)	Electron microscopy	Rat/striatum	S1 Percoll fraction 4	905 ± 12 nm
Hollingsworth et al. (1985)	Electron microscopy	Guinea pig/cortex	Filtration synaptoneurosomes	560 ± 15 nm
Hollingsworth et al. (1985)	Light microscopy	Guinea pig/cortex	Filtration synaptoneurosomes	∼600 nm
Williams et al. (2009)	Phase contrast microscopy	Human/cortex	Filtration synaptoneurosomes	300 ± 700 nm
Fernández-Busnadiego et al. (2013)	Cryo-electron tomography	Mouse/cortex	Percoll fraction 3 + 4	500 ± 1000 nm

In contrast, another widely adopted protocol (Gylys and Bilousova, 2017) implements FSC triggering and specifically analyzes only events in the FSC range defined by 0.5- to 1.5-µm PS beads. Based on the high percentage of events that carry immunofluorescent signal for synaptic markers, these events are assumed to represent single synaptosomes and material below 500-nm PS beads is regarded as debris (Gylys et al., 2004). One study analyzed “large synaptosomes,” events in an FSC range defined by 1.5- to 4.5-µm PS beads (Gylys et al., 2000). We found that the FSC range above 2-µm PS beads includes particles as large as neuronal nuclei (Extended Data Fig. 1-1*C*). To our knowledge, the only single synaptosomes approaching this size are derived from mossy fiber terminals of the hippocampus and cerebellum, and accordingly these synaptosomes sediment along with nuclei in the P1 fraction (Taupin et al., 1994; Terrian et al., 1988). In our hands, the FSC region above 1.3-µm silica beads (which would fall below both 1.5- and 4.5-µm PS beads) represents <5% of all events in the P2 sample (Fig. 6*C*) and contains the highest frequency of false double-positive events (Figs. 6*E–H*, 7*C*). Our results strongly suggest that a large fraction of events in these regions are synaptosome-containing aggregates, while single synaptosomes are often found below 500-nm PS beads and the FSC noise threshold.

### Fluorescence triggering and dilution to control for coincidence

Maintaining an appropriately low sample concentration is critical to prevent coincident particle detection in submicron flow cytometry experiments. It has been suggested that coincidence is especially prominent when operating in FSC-trigger mode (Nolan and Stoner, 2013), which might explain the variations in FSC and FM4-64 fluorescence we observed with dilution in FSC-trigger mode on both cytometers (Fig. 2*C*,*D*). Importantly, we found that FSC-trigger mode substantially underestimates the true event rate in P2 samples on both cytometers. In such a situation, undetected particles in the sample volume associated with a detectable particle can contribute to the fluorescence measurements and/or be sorted in the same drop (Libregts et al., 2018). Thus, with FSC triggering at high event rates, one cannot determine if sorted material accurately reflects the detection and sorting of single particles. Although we found that aggregation was the major source of double-positive events in our samples, we did not conduct these assays using FSC-trigger mode or at high event rates. Serial dilutions and fluorescence triggering should be conducted in all submicron flow cytometry experiments to avoid coincidence.

### Detection of coincidence and aggregation

To our knowledge, our study is the first to systematically address coincidence and aggregation in flow cytometric analysis of synaptosomes (Fig. 3). We chose a strategy analogous to a “mixed-species” experiment commonly employed to validate single cell RNA-sequencing systems, where a mixture of mouse and human cells is analyzed to quantify cell-doublet rates (Klein et al., 2015; Macosko et al., 2015). Our dye labeling strategy is also employed in high-throughput flow cytometry studies, where cells from different conditions are given non-transferable fluorescent barcodes before mixing, staining, and acquisition in a single batch (Krutzik et al., 2011). Although the cellular fluorescent barcodes are decoded with high accuracy in these protocols, even when multiple barcodes are encoded by discrete concentrations of a single dye (Krutzik and Nolan, 2006), our results demonstrate that synaptosome preparations do not behave similarly to cells in these experiments. Determining the extent of single-particle detection in submicron flow cytometry experiments is a challenging task. Image cytometry, which combines confocal microscopy with the fluidics of a cytometer, is powerful in this regard since images of each event can be manually examined after acquisition. However, these instruments require extensive optimization and are not widely available to all labs. The fluorescent labeling and mixing assay we describe here can be implemented on most conventional flow cytometers and should be broadly useful to the microparticle flow cytometry community.

### Distinguishing between coincidence and aggregation

As we were unable to reduce the frequency of false double-positive events with extensive dilution and FM triggering of our P2 mixtures (Fig. 4*C*,*D*), we began to suspect aggregation as the source of these events. Aggregated particles would behave as “single” particles in terms of dilution and coincidence, but nonetheless present serious problems for both cellular and submicron flow cytometry analysis. The large scatter signals produced by cells result in abnormal width/height versus area pulse profiles, which can be used to remove cell-doublet events in conventional flow cytometry experiments. To date, we are unaware of any study demonstrating that this gating strategy works on submicron particles, and our results strongly suggest it does not (Extended Data Fig. 3-1*C*). We further demonstrated that double-positive events in our P2 mixtures can be enriched by sorting (Fig. 5*A–C*), while fluorescent PS bead double-positive events are depleted by sorting. These results are highly consistent with our dilution series and provide further evidence that the dominant source of double-positive events in P2 mixtures is aggregation. Combined with our fluorescent labeling and mixing assay, comparison to fluorescent PS bead mixtures in dilution series and sorting experiments provides a general strategy to determine the extent of coincidence versus aggregation in any sample type.

### Synaptosome aggregation

Single events comprised of aggregated cell-derived microparticles have been definitively identified using image cytometry (Erdbrügger et al., 2014). It is unclear how often and under what conditions cell-derived microparticles aggregate, but one can speculate that their endogenous function within physiologic bodily fluids would make them somewhat resistant to aggregation. In contrast to cell-derived microparticles, presynaptic nerve terminals do not exist as soluble particles *in vivo*, and the aggregation of synaptosomes in ionic media (saline-based solutions such as PBS) is a well-described phenomenon. Gray and Whittaker (1962) analyzed the minimum concentration of various electrolytes required to induce aggregation of particles in the P2 and sucrose-gradient synaptosome fractions. They describe how the particles behave as though negatively charged, since divalent and trivalent cations are particularly effective in causing aggregation. However, the minimum concentration for sodium chloride was 20 mM (Gray and Whittaker, 1962), far below the 137 mM present in PBS. Others encountered such aggregates using a filtration procedure to purify “synaptoneurosomes” from brains homogenized in saline (Hollingsworth et al., 1985).

Most synaptosome flow cytometry studies have employed ionic media for incubation, washing, and sample analysis, while assuming that each event generated by the flow cytometer represents a single synaptosome (Gylys et al., 2000, 2004; Fein et al., 2008; Postupna et al., 2014; Prieto et al., 2017). Studies of cell-derived microparticles have clearly demonstrated situations where this assumption breaks down (Erdbrügger et al., 2014; Libregts et al., 2018), and this issue was recently brought forth in synaptosome flow cytometry (see comments on Prieto et al., 2017). The centrifugation and time-lapse variations of our fluorescent mixture experiments provide further insight into particle aggregation in P2 samples (Fig. 5*D*). Most strikingly, we found that centrifugation and resuspension of the single-labeled mixture dramatically increases the frequency of double-positive events. This finding strongly suggests that resuspension of the P2 pellet does not completely eliminate particles that aggregate during centrifugation. We also observed a steady increase in the frequency of double-positive events over time with or without centrifugation (Fig. 5*D*), suggesting that aggregation is an ongoing process in P2 sample mixtures. These results also further argue against coincidence as a cause of these double-positive events, as coincidence would not be expected to increase over time. Although washing steps are critical to these experiments, our data suggest that repeated centrifugation will bias the sample composition. The enhanced relative abundance of larger, brighter particles following centrifugation in PBS (Fig. 8*D–F*), especially at low speeds, is consistent with previous work (Gray and Whittaker, 1962) and provides further evidence that many of these high FSC events are aggregated particles. Future studies should carefully consider the effects of duration, speed, and number of centrifugations on downstream experimental results.

### Reducing synaptosome aggregation

Analogous to the microscopy results of other groups (Choi et al., 2009; Daniel et al., 2012), we found that SET buffer significantly reduced the frequency of double-positive events in our P2 mixtures, especially in the higher FSC gates (Fig. 6*H*). However, calcein-labeling efficiency was also lower in SET buffer (Extended Data Fig. 6-1*C*) and SET would not be compatible with many physiologic assays (Choi et al., 2009). We found that conducting the labeling procedure in PBS and switching to SET buffer for flow cytometry also reduced the double-positive event frequency, although the effect was smaller than for SET alone (Fig. 6*H*). Our flow cytometry assay provides a quantitative measure (albeit an underestimate) of synaptosomal aggregation in each buffer condition and should be useful to monitor the impact of future protocol improvements. Even without the fluorescent mixture assay, we observed that SET buffer reduces the relative abundance of particles in the highest FSC regions most likely to contain aggregates (Fig. 8*D–F*). Anecdotally, we noticed that the P2 pellet disperses relatively easily in SET buffer compared to PBS. Based on the findings of Gray and Whittaker (1962), we suspect that removing cations reduces aggregation by allowing the negatively charged neuronal membranes to repel each other.

### Pitfalls in immunophenotyping of synaptosomes

The heterogeneity of presynaptic nerve terminals, including specific cases of neurotransmitter co-release (Hnasko and Edwards, 2012; Vaaga et al., 2014), makes high-throughput immunophenotyping of synaptosomes by neurotransmitter content an appealing direction for synaptosome flow cytometry. Indeed, Gajera et al. (2019) have recently demonstrated high-dimensional analysis of human synaptosomal preparations using mass cytometry. Our results suggest that the presence of synaptosomal aggregates is a serious problem for such experiments, especially in the top two FSC ranges in our study (higher than 880-nm silica beads; Fig. 7*C*). We found implausibly high co-labeling of VGAT, VGLUT1, and VMAT2 in our P2 samples (Extended Data Fig. 7-2), and the increasing frequency of double-positive and triple-positive events observed across FSC gates corroborates the analogous finding in our fluorescent mixture assay (Figs. 6*D–F*, 7*C*). These findings further support our conclusion that a high percentage of events in this FSC range are synaptosome-containing aggregates. As expected based on synapse abundance, VGAT+/VGLUT1+ double-positive events were observed most frequently (Extended Data Fig. 7-1*C*,*D*). Similarly, Biesemann previously demonstrated co-enrichment of VGAT and VGLUT1 by sorting VGLUT1 fluorescent events between 0.75- and 1.5-µm beads (Biesemann, 2010; his Results 3.4, Fig. 14, pp 86–87). Western blotting of the sorted material revealed enrichment of VGAT and myelin proteolipid protein (PLP), proteins that should be depleted in a sort of pure VGLUT1+ synaptosomes. After implementing FM triggering and sorting only VGLUT1 fluorescent events below the FSC noise threshold, the authors demonstrated unprecedented purity of sorted VGLUT1 synaptosomes with the expected depletion of VGAT and other glial markers (Biesemann et al., 2014). Overall, our results are highly consistent with their conclusion that the previously sorted events were aggregates containing at least one glutamatergic synaptosome (Biesemann, 2010; his Results 3.4, p 86).

As others have pointed out (Erdbrügger et al., 2014), immunophenotyping of submicron particles can result in detection, but not necessarily accurate quantification. Unfortunately, we found that SET buffer was less effective in reducing false double-positive and triple-positive immunostained events (Fig. 7*C*) compared to those observed in the fluorescent mixture assay (Fig. 6*H*). Since resuspension of the P2 pellet is unlikely to completely eliminate aggregates, we suspect that some aggregated particles are cross-linked during formaldehyde fixation before immunostaining. Accordingly, we found that single-positive event frequency was generally ∼90% below the FSC noise threshold in immunostained samples (Fig. 7*C*), while the single-positive frequency in this region was generally ∼97% in the calcein mixtures (Fig. 6*D*). Although this suggests that some of the double-positive events in the immunostained samples are single synaptosomes with true colocalization (e.g., VGAT+/VGLUT1+ nerve terminals in cortical Layer V; Fattorini et al., 2009), distinguishing such events from aggregates would require extensive experimental validation. Flow cytometry studies claiming to study single synaptosomes often report a high-degree of co-labeling for markers that are expected to colocalize, such as VGLUT1, PSD95, synaptophysin, SNAP25, etc. (Gylys et al., 2004; Prieto et al., 2017). Such data does not provide evidence that the detected particles are single synaptosomes, since synaptosome-containing aggregates would be expected to yield similar, if not even higher co-labeling for these markers. Instead, we propose that such studies should explicitly report the co-labeling of neurotransmitter-specific markers (i.e., VGLUT1, VGAT, etc.) that should be mutually exclusive for most single synaptosomes. As suggested previously (Nolan and Stoner, 2013), claims of co-localization should be supported by direct evidence that coincidence and aggregation are not contributing to the measurement.

### Challenges and future directions

Here, we highlighted a number of technical challenges that hinder the detection and analysis of single particles in synaptosome flow cytometry. In the absence of new purification methods, synaptosome preparations will inevitably be contaminated with membranes, myelin, and mitochondria. Currently, it seems that transgenic mice harboring fluorescent protein reporters are required for sorting, since tractable surface markers are limited and most downstream assays are not compatible with fixation and immunostaining of cytoplasmic proteins. A sorting protocol for VGLUT1^Venus^ synaptosomes has described by Herzog and colleagues (Biesemann et al., 2014; Luquet et al., 2017; see also Poulopoulos et al., 2019 for growth cone sorting) and should be readily adaptable for other fluorescent proteins and synapse types. However, if immunostained synaptosome events are the analytical endpoint for an experiment, the methods described in our study will minimize the effects of coincidence and aggregation on the analysis of single synaptosomes. Such procedures will need to be routinely performed on each cytometer, and confirmation of accuracy by microscopy seems prudent. We remain optimistic that future advances in instrumentation, reagents, and sample preparation will obviate the need for such extensive QC. Despite current limitations, we expect synaptosome flow cytometry studies to continue enhancing our understanding of synaptic biology in health and disease.

## Synthesis

Reviewing Editor: Jeffery Twiss, University of South Carolina Decisions are customarily a result of the Reviewing Editor and the peer reviewers coming together and discussing their recommendations until a consensus is reached. When revisions are invited, a fact-based synthesis statement explaining their decision and outlining what is needed to prepare a revision will be listed below. The following reviewer(s) agreed to reveal their identity: JP Nolan.

Synaptosome preparations have been a key approach for biochemical and molecular studies of individual synapses. Obtaining pure synaptosomes is a biochemical and technical challenge. Many independent studies have employed flow cytometry to overcome the biochemical challenge, but detection artifacts often complicate these. The current manuscript uses two flow cytometry instruments to test a variety of experimental conditions to layout the pitfalls and challenges in purifying synaptosomes. The authors address some of the major shortcomings of previous efforts to measure synaptosomes using flow cytometry, particularly the issues of detection trigger channel and the coincident occurrence of multiple particles within the laser beam, which confounds data interpretation. Overall the manuscript is written for non-experts, with clear and detailed methods section, and inclusion of their measurement criteria, so it should be of high interest to eNeuro's readers and useful for the neuroscience community. It is also is well illustrated and data clearly presented, which can sometimes be a challenge in a technique manuscript. Despite these clear strengths, the reviewers noted several issues that would need to be addressed before the manuscript can be further considered for publication.

This manuscript describes the flow cytometric analysis of particles in preparations of mouse brain homogenates, including pellets after centrifugation that are identified and “crude nuclei” and “crude synaptosomes.” The authors compare the results of FSC trigger-based event detection with FM1-64 fluorescence trigger-based event detection and find improved detection of particles with fluorescence triggering. They assess the hallmarks of coincidence by measuring serial dilutions of sample and find more coincidence with FSC triggering compared to fluorescence. They explore the occurrence of coincidence and aggregation in samples comprised of differently labeled particle preparations. Finally, they investigate the staining of the detected particles with antibodies against different neurotransmitter reporters.

1. Coincidence can often be revealed by anomalous behavior in a sample dilution series, where single particle measurements are expected to result in a proportional decrease in measured events (and event rate) while the brightness of the measured events should not change. Figure 2 and Figure ED2 present the results of such an analysis for the Influx and Fortessa cytometers, respectively and show clear evidence of coincidence (large changes in population median intensities with dilution) for scatter-based measurements in both instruments. Fluorescence trigger-based measurements do not show such large changes, although the measured intensities are so low and close to background that decreases would be hard to measure. Nonetheless, these data nicely illustrate this common artifact, as the superiority of fluorescence-triggered particle measurements over scatter-triggered analysis.

2. The authors use beads and synaptosome preps stained individually and then mixed for analysis to illustrate the occurrence of coincidence and aggregation, two phenomena that can be hard to distinguish. They show that coincidence can be ameliorated by dilution of sample, but that aggregation cannot. They present data (Figure 5) that suggests that aggregation increases over time and that centrifugation (using their standard method of 10K xg for 5 minutes) increases the fraction of aggregates, and that the use of a non-ionic sucrose buffer also reduced aggregation compared to PBS.

3. This analysis is plausible, but the data are noisy and highly normalized. It is preferable to present the data as raw numbers or concentrations so that the reader can evaluate the variability in the data. Presentations of measurements as % Positive are useful as long as the denominators is well behaved, otherwise it can be misleading.

4. Related to the knowledge of the denominator (which is, presumably the total particle/synaptosome concentration) is the uncertainty introduced by repeated centrifugation. The sequential centrifugation steps to produce the P1 pellet and S1 supernatant (1000 xg, 10 min), and then the P2 synaptosome pellet (10,000 xg, 20 min), appear straightforward enough, but a look at the prior work cited in the paper reveals differences (e.g. the second spin in Biesemann is 12,500 for 15 minutes) that may or may not be important. It would be useful if the authors could justify these differences and comment their effect on the present results and implications for standardization in the field. Moreover, for many of the experiments here, and the immunofluorescence results in particular, it appears that the samples were repeatedly subjected yet another centrifugation protocol (10,000 xg, 5 min). This reviewer can't help wondering what additional fractionation has occurred during these steps. What is the recovery after these additional wash steps (noting that his would be clear if the data were presented as absolute number/concentration, rather than a normalized % positive events)?

5. The immunostaining protocol raises additional questions. The authors choose to use an indirect immunostaining approach, with an initial incubation with primary antibody in 0.1% tween followed by two centrifugation (10K xg, 5 min)-based washes followed by incubation with fluorescence-labeled secondary antibodies (again in 0.1% Tween) followed by two more wash steps. First, Tween 20 is a surfactant and known to solubilize vesicles, so it appears that the authors are detecting a surfactant-resistant set of particles/epitopes, in addition to selecting a large/high density set for particles by repeated short centrifugations. It would clearly have been preferable to block with a protein such as BSA or irrelevant immunoglobulin and to stain with a direct-labeled primary antibody so as to avoid the multiple centrifugation-based washed steps, whose impact on the resulting measured particle is neither known nor discussed.

6. Further, flow cytometry best practices dictate that, where possible, fluorescence measurements should be calibrated using commercially available intensity standard beads and expressed in standards MESF (mean equivalent soluble fluorochromes). In this case, Alexa 488 (used for secondary immunostaining) is sufficiently similar to fluorescein (similar spectra and within a factor or ~2 or so in brightness) that calibrating and expressing the A488 immunostaining brightness in FITC MESF equivalents would be useful for others who wish to reproduce or compare the authors' measurements.

## Author Response

We thank the reviewers for their excellent suggestions, which were instrumental in revising our work for enhanced clarity and impact. As you will see below, the reviewers' comments also provided impetus for a new experiment that we feel significantly strengthens the manuscript. We have highlighted changes within the manuscript file in red font, and we also included the changes relevant to each comment below.

1. Coincidence can often be revealed by anomalous behavior in a sample dilution series, where single particle measurements are expected to result in a proportional decrease in measured events (and event rate) while the brightness of the measured events should not change. Figure 2 and Figure ED2 present the results of such an analysis for the Influx and Fortessa cytometers, respectively and show clear evidence of coincidence (large changes in population median intensities with dilution) for scatter-based measurements in both instruments. Fluorescence trigger-based measurements do not show such large changes, although the measured intensities are so low and close to background that decreases would be hard to measure. Nonetheless, these data nicely illustrate this common artifact, as the superiority of fluorescence-triggered particle measurements over scatter-triggered analysis.

We thank the reviewer for these comments and agree that fluorescence-triggering is superior for small particle flow cytometry.

2. The authors use beads and synaptosome preps stained individually and then mixed for analysis to illustrate the occurrence of coincidence and aggregation, two phenomena that can be hard to distinguish. They show that coincidence can be ameliorated by dilution of sample, but that aggregation cannot. They present data (Figure 5) that suggests that aggregation increases over time and that centrifugation (using their standard method of 10K xg for 5 minutes) increases the fraction of aggregates, and that the use of a non-ionic sucrose buffer also reduced aggregation compared to PBS.

We thank the reviewer for this concise summary.

3. This analysis is plausible, but the data are noisy and highly normalized. It is preferable to present the data as raw numbers or concentrations so that the reader can evaluate the variability in the data. Presentations of measurements as % Positive are useful as long as the denominators is well behaved, otherwise it can be misleading.

We agree that raw event counts are ideally preferable, but practically, such data would only be intelligible if precisely the same number of events were collected per sample. When quantifying a particular event type, the vast majority of flow cytometry studies normalize to the total event count within each sample. Even this is often not sufficient, as variability in the sample composition (i.e. ratio of cells to debris amongst all events) can obscure meaningful comparisons. In many cases, the quantification of an event type is most meaningful when expressed as a percentage of a specific parent population. For example, the fractional abundance of VGLUT1+ synaptosomes amongst all particles might vary across brain regions, but the fraction of VGLUT1+ synaptosomes that stain for PSD95 might remain constant. Similarly, the number of double-positive events in our fluorescent mixture assay depends on 1) the number of total events as well as 2) the fractional abundance of fluorescently-labeled particles in the sample. We therefore express the double-positive events as a percentage of all fluorescent events for each sample. Because we do see variability in the denominator (see Figure 6-1C for calcein labeling, which is the denominator for Figure 6H), the metric of aggregation in fact requires within-sample normalization. To clarify this point in the ‘Multicolor Labeling and Mixing Assays’ section of the Materials and Methods, we now write on lines 255-258:

“For fluorescence mixture assays, we observed variability in the calcein labeling efficiency across samples. Since only fluorescent particles are eligible to report on aggregation, we normalize each fluorescent event type (i.e. single-positive/double-positive) to all fluorescent events within each sample.”

We recognize that there is variability across samples in each buffer condition. We specifically used box and whisker plots for this reason, and we clearly state that sucrose buffer reduces, but does not eliminate, double-positive events in the calcein mixture assay. However, across 8-10 samples per buffer condition, two-way ANOVA revealed a highly significant effect of buffer on the normalized double-positive event frequency, an effect that was especially prominent in the higher FSC regions where aggregation is more prevalent (Figure 6H). We were also especially careful to point out that the effect of sucrose buffer was much more modest for immunostaining experiments (Figure 7C). Finally, we have also added new data on particle abundance before and after centrifugation in PBS and sucrose buffer that provides further support of increased aggregation in PBS (see comment #4 below).

With regards to particle concentrations, we maintain the lowest possible sample pressure in all experiments and dilute samples until low event rates are achieved (excluding dilution series experiments). While this ensures that a reasonably similar range of sample concentrations are analyzed across experiments, we agree that measurement of particle concentrations across the range of our dilution series (Fig. 2 and 4) would be of broad interest. We used volumetric counting beads (see Materials and Methods, lines 281-293) to measure the particle concentration based on FM4-64-triggered events across a typical dilution series. These data are now presented in Figure 2-1C. We now write on lines 441-444 of Results:

“Since sample flow rates and event rates may vary across cytometers, we also measured the absolute particle concentration across a typical dilution series on the Influx using volumetric count beads (Figure 2-1C). We found that the most linear part of the event rate curve was for samples below ~1000 particles/µL.”

4. Related to the knowledge of the denominator (which is, presumably the total particle/synaptosome concentration) is the uncertainty introduced by repeated centrifugation. The sequential centrifugation steps to produce the P1 pellet and S1 supernatant (1000 xg, 10 min), and then the P2 synaptosome pellet (10,000 xg, 20 min), appear straightforward enough, but a look at the prior work cited in the paper reveals differences (e.g. the second spin in Biesemann is 12,500 for 15 minutes) that may or may not be important. It would be useful if the authors could justify these differences and comment their effect on the present results and implications for standardization in the field. Moreover, for many of the experiments here, and the immunofluorescence results in particular, it appears that the samples were repeatedly subjected yet another centrifugation protocol (10,000 xg, 5 min). This reviewer can't help wondering what additional fractionation has occurred during these steps. What is the recovery after these additional wash steps (noting that his would be clear if the data were presented as absolute number/concentration, rather than a normalized % positive events)?

We agree that particle abundance following repeated centrifugations warranted further study and now provide new experimental evidence to address this point.

The first spin after shearing homogenization in most synaptosome protocols is designed to sediment nuclei and large debris, and is generally between 700-1000xg for 10-15min. While the first spin is fairly standardized across most protocols, the second spin is more diverse, which we have seen range from 7,000-20,000xg for 15-30min. One important aspect to consider here is the experimental goal. For investigators conducting protein-based bulk synaptosome experiments, generally a high starting volume of homogenate (often &gt;10mL) combined with further purification of the P2 pellet using either a sucrose, Percoll, or Ficoll gradient procedure may require a higher speed/longer ‘P2 spin’ in order to maximize yield. Similarly, we wanted to maximize yield for our initial preparation of the P2 fraction prior to aliquoting in small samples for cryopreservation, and we thus chose 10,000xg for 20min.

For flow cytometry experiments involving repeated washing/centrifugation steps on small scale samples (handled in 1.5mL tubes), shorter and slower spins are generally used. Previous synaptosome flow cytometry studies have used washing centrifugation speed/times as low as 2,000xg/4min (Gylys et al., 2004) or 2,500xg/5min (Prieto et al., 2017) without any data on particle recovery. We opted to compromise at 10,000xg/5min in order maximize recovery while reducing the length of experiments.

Another key aspect of particle recovery during centrifugation is the buffer employed. Gray and Whittaker (1962) reported increased recovery of acetylcholine in the P1 fraction when using saline instead of sucrose buffer, which was interpreted as aggregation in saline increasing the effective size of synaptosomes (and thus sedimentation at lower speed). Inspired by this experiment, we used flow cytometry with volumetric count beads as a readout for particle abundance of P2 samples after 5-minute spins of varying relative centrifugal force in PBS and sucrose buffers (see new Figure 8A, and Materials and Methods, lines 281-293).

Repeated centrifugations did incur particle losses in both PBS and sucrose buffer (see new Figure 8-1C), which seems to be an unavoidable aspect of these protocols. However, our results indicate that PBS substantially biases samples towards larger particles that are likely to be aggregates, while reducing total particle abundance when compared to sucrose buffer (see new Figure 8B-D). The bias towards aggregates in PBS is stronger when lower centrifugation speeds are used (see new Figure 8E-F). These data add further support to the model of increased particle aggregation in PBS and suggest that low-speed centrifugations should be avoided. We now write in the new ‘Effects of Buffer and Centrifugation on Particle Abundance’ section of the Results (lines 697-736):

“Given the increased frequency of aggregates after centrifugation (Figure 5D), in higher FSC regions (Fig. 6D-F), and in PBS (Fig. 6H), we wondered whether buffer and centrifugation might interact to bias sample composition towards aggregates following repeated centrifugations. Indeed, Gray and Whittaker (1962) previously reported increased recovery of acetylcholine in the low-speed P1 pellet when using saline instead of sucrose buffer, which was interpreted as aggregation in saline increasing the effective size of synaptosomes (and thus sedimentation at lower speed). To assess whether multiple centrifugation steps might bias particle recovery in PBS vs. SET, we conducted two sequential 5-minute centrifugations of P2 samples in PBS or SET buffer at varying speeds and used flow cytometry with absolute counting beads to determine the particle concentration of the pellets and supernatants (Figure 8A). We found that under carefully controlled conditions with the absolute counting beads, PBS reduced particle abundance by ~3-fold prior to any centrifugation (Figure 8B). It is possible that certain particles in the P2 sample are susceptible to destruction in PBS, but several features of the data suggest that aggregation contributes to the reduction in particle counts. First, the median fluorescence intensity of membrane staining by FM4-64 was higher in PBS (Figure 8B), which was due to increased relative abundance of brighter, larger events with higher FSC (Figure 8C-D). In line with the findings of Gray and Whittaker (1962), we found that these ‘FSC High’ particles were more efficiently pelleted in PBS at lower relative centrifugal force (2500-5000xg) compared to SET buffer (Figure 8D). The relative abundance of these particles was dramatically increased in the two pellets, P2-1 and P2-2, compared to the input sample and two supernatants (Figure 8E). The relative abundance of the large particles in PBS samples was highest in 2500xg pellets and lowest in 10000xg pellets, while no clear relationship between relative abundance and centrifugation speed was observed in SET buffer (Figure 8F). A slight increase in these ‘FSC High’ particles was observed when comparing spin 1 vs. spin 2 for both buffers, but this effect was modest compared to the increase in PBS vs. SET (Figure 8F).

As expected, the overall efficiency of particle recovery in the pellet of each spin increased with centrifugation speed, with the exception of the first spin in PBS (Figure 8-1A-C). Similar to the input samples, it appears that a combination of aggregation and/or destruction contribute to the loss of particle counts in the first PBS spin, although the recovery in the second spin was higher than in SET buffer. Critically, the first 5-minute spin at 2500-5000xg in either buffer leaves over half the P2 particles in the supernatant (Figure 8-1D). Collectively, these data suggest that P2 samples do undergo a certain degree of further fractionation during repeated centrifugation-based wash steps. Although some centrifugation bias is inevitable regardless of buffer and centrifugation speed, our data suggest that lower centrifugation speeds and ionic buffers will bias the overall sample composition towards larger particles that are more likely to be aggregates.”

And in Discussion (lines 883-889 and lines 902-904, respectively):

“Although washing steps are critical to these experiments, our data suggest that repeated centrifugation will bias the sample composition. The enhanced relative abundance of larger, brighter particles following centrifugation in PBS (Fig. 8D-F), especially at low speeds, is consistent with previous work (Gray and Whittaker, 1962) and provides further evidence that many of these high FSC events are aggregated particles. Future studies should carefully consider the effects of duration, speed, and number of centrifugations on downstream experimental results.”

“Even without the fluorescent mixture assay, we observed that SET buffer reduces the relative abundance of particles in the highest FSC regions most likely to contain aggregates (Fig. 8D-F).”

We thank the reviewer for this insightful comment: the centrifugation recovery data should be of broad interest in this area, and also provide orthogonal confirmation of increased particle aggregation in ionic buffer.

5. The immunostaining protocol raises additional questions. The authors choose to use an indirect immunostaining approach, with an initial incubation with primary antibody in 0.1% tween followed by two centrifugation (10K xg, 5 min)-based washes followed by incubation with fluorescence-labeled secondary antibodies (again in 0.1% Tween) followed by two more wash steps. First, Tween 20 is a surfactant and known to solubilize vesicles, so it appears that the authors are detecting a surfactant-resistant set of particles/epitopes, in addition to selecting a large/high density set for particles by repeated short centrifugations. It would clearly have been preferable to block with a protein such as BSA or irrelevant immunoglobulin and to stain with a direct-labeled primary antibody so as to avoid the multiple centrifugation-based washed steps, whose impact on the resulting measured particle is neither known nor discussed.

We emphasize that we did not intend to optimize immunostaining in this manuscript. Rather, our immunostaining approach was based on previous work (Gylys et al., 2004, Postupna et al., 2014, Prieto et al, 2017). We note that in all these studies, the preparations are thoroughly fixed with paraformaldehyde prior to permeabilization: 4% PFA for 15-20 min at 22{degree sign}C in our study and in Prieto et al. (2017), or 0.25% PFA for 60 min at 4{degree sign}C in Gylys et al. (2004) and Postupna et al. (2014).

In general, it seems that presynaptic terminals contain a sufficient density of proteins such that after formaldehyde fixation the crosslinked network confers stability against solubilization of cytoplasmic contents. This is supported by the fact that fixed brain tissue sections retain presynaptic vesicle proteins even after extensive incubation in harsh solvents such as methanol or Triton X-100. The same is true for purified VGLUT1-mVenus synaptosomes immunostained on glass using PFA/Triton X-100 fixation/permeabilization conditions (Biesemann et al., 2014). Prieto et al. (2017) also demonstrate staining of synaptosomal markers by flow cytometry using P2 samples fixed with PFA followed by methanol, which is quite harsh on lipids. Finally, previous synaptosome flow cytometry studies have all used Tween20 for permeabilization and/or washing (Gylys et al., 2004, Postupna et al., 2014, Prieto et al, 2017). Therefore, we feel our use of Tween20 is justified in order to best compare to previous studies. We agree that the choice of detergent and its concentration during immunostaining merit further investigation, but we feel that optimization of various permeabilization conditions is beyond the scope of the present work.

With regards to blocking and antibody staining, we note that we did indeed include 5% BSA in the blocking buffer (along with 0.1% Tween20) which was present during permeabilization, primary antibody staining, and secondary antibody staining. We agree that repeated centrifugation-based washes are a disadvantage of the immunostaining protocol, as our data in Fig. 5D and new Fig. 8 show that this is likely to bias towards aggregates. However, we had previously conducted a ‘no wash’, direct immunostaining protocol using Fab-AlexaFluor488-labeled primary antibody (using Invitrogen Zenon technology) followed by dilution and flow cytometry without centrifugation, as the reviewer suggests. As shown below for GluR1 (a subunit of AMPA glutamate receptors), the results are similar, but the direct protocol resulted in a lower signal compared to the indirect, AlexaFluor488-conjugated secondary protocol.

We therefore chose to use an indirect approach for three reasons: 1) precedent in previous studies (Postupna et al., 2014, Prieto et al., 2017), 2) commercially available fluorophore-conjugated primary antibodies against synaptic proteins are limited, and 3) extra signal amplification gained by the secondary antibody step is desirable for these small particles. While optimizations of immunostaining protocols are worthy of further study, such improvements will require extensive work that is not directly relevant to the central focus of this manuscript, which is flow cytometry particle detection artifacts.

6. Further, flow cytometry best practices dictate that, where possible, fluorescence measurements should be calibrated using commercially available intensity standard beads and expressed in standards MESF (mean equivalent soluble fluorochromes). In this case, Alexa 488 (used for secondary immunostaining) is sufficiently similar to fluorescein (similar spectra and within a factor or ~2 or so in brightness) that calibrating and expressing the A488 immunostaining brightness in FITC MESF equivalents would be useful for others who wish to reproduce or compare the authors' measurements.

This is another excellent suggestion, and we have conducted immunostaining of VGAT with AlexaFluor488-conjugated anti-mouse secondary antibody (as in Fig. 7) in conjunction with commercially available Alexa488 MESF calibration beads (see Figure 7-1E, and Materials and Methods, lines 295-309).

Expressing the ‘immunostaining brightness’ of P2 samples in Alexa488 MESF is not simple, because particle fluorescence is directly related to FSC intensity regardless of the immunostaining procedure. Thus, many ‘immunopositive events’ at a given FSC intensity would exhibit green fluorescence intensity less than or equal to ‘immunonegative events’ at a higher FSC intensity nearby (see https://www.biolegend.com/newsdetail/1182/ for a nice visualization of this phenomenon, where FL2 would represent FSC in our case). We therefore compared the Alexa488 MESF intensity of immunopositive/negative gated events within each FSC gate (see Materials and Methods, lines 295-309). Shown in Figure 7-2F are the median fluorescence intensity and robust standard deviation in Alexa488 MESF units for VGAT immunopositive and immunonegative events in each FSC gate. These data are also included in Table 7-2. We now write in Results, on lines 686-695:

“Although scatter measurements of submicron particles may vary dramatically across cytometers, fluorescence measurements can be readily calibrated. We determined the fluorescence intensity of VGAT/anti-mouse Alexa488 immunostaining in molecules of equivalent soluble fluorophore (MESF) using calibrated bead standards (Fig. 7-1E). Because particle fluorescence is directly related to FSC intensity regardless of the immunostaining procedure, many immunopositive events at a given FSC intensity would exhibit fluorescence intensity less than or equal to immunonegative events at a higher FSC intensity nearby. We therefore analyzed the median fluorescence intensity of gated immunopositive and immunonegative events within each FSC gate (Fig. 7-1F). Numerical MESF data are also included in Table 7-2, which should facilitate comparison across laboratories.”
