# Supplementary figures and images for: Critical Analysis of Particle Detection Artifacts in Synaptosome Flow Cytometry
Source: eNeuro. 2019 Jun 4;6(3):ENEURO.0009-19.2019. doi: 10.1523/ENEURO.0009-19.2019 (PMC6565374; doi:10.1523/ENEURO.0009-19.2019)

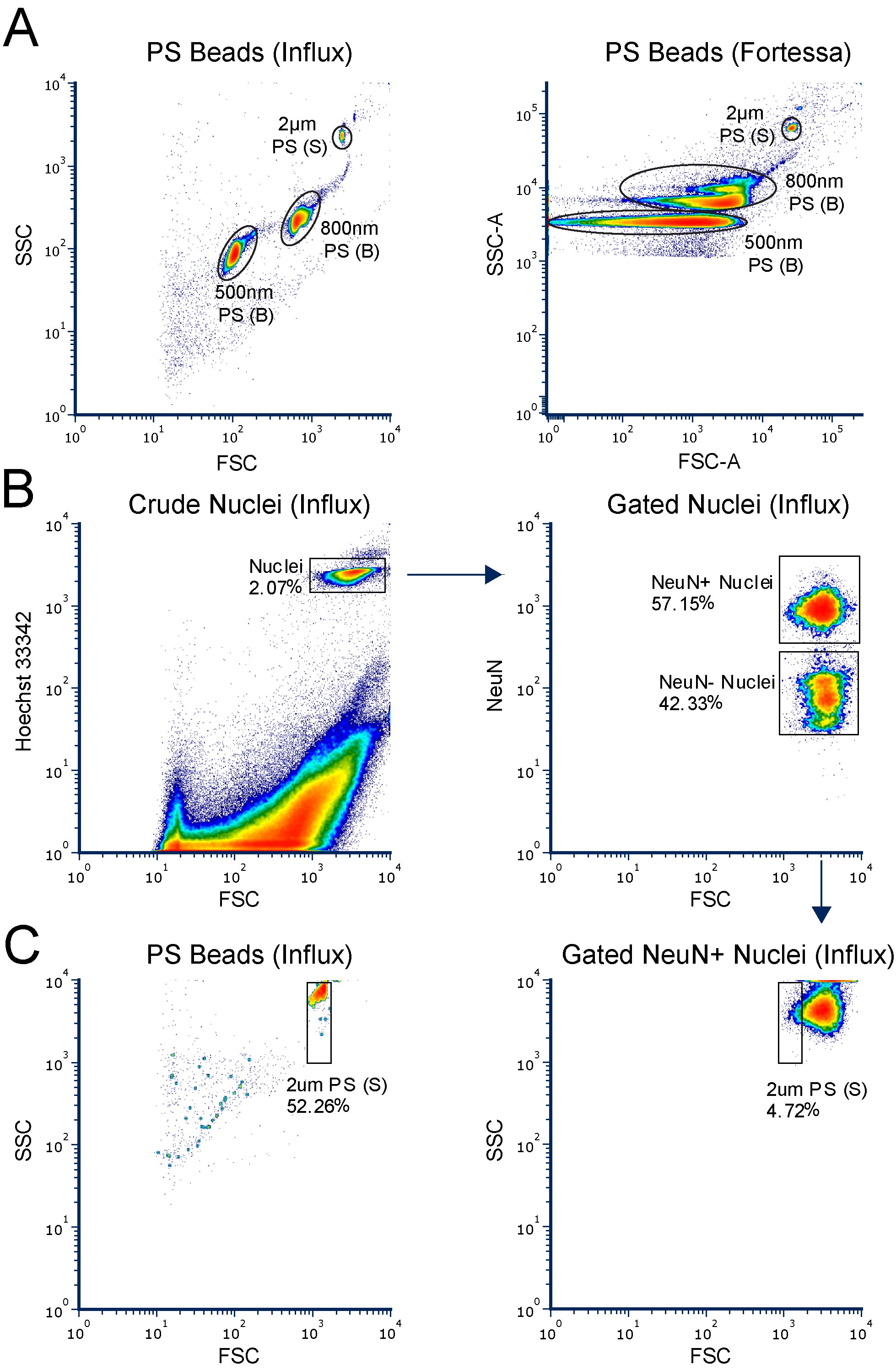

Supplement: Extended Data Figure 1-1 — Comparison of FSC signal from neuronal nuclei and 0.5-, 0.8-, and 2.0-µm PS beads. We note that in these experiments, the FSC detector voltage had to be lowered in order to allow the 2.0-µm PS to fall within the detectable range; the FSC axis is therefore substantially different from all other plots in this study. A, Mixture of fluorescent PS beads (Bangs Labs; B, or Spherotech; S) detected in FSC-trigger mode on the Influx (left) and SSC-trigger mode on the Fortessa (right). We note that the lowered FSC voltage on the Fortessa resulted in a significant amount of FSC noise. B, Crude nuclei detected in FSC-trigger mode on the Influx. Left, Hoechst33342 fluorescent staining of DNA identifies nuclei apart from all other particles. Right, Gated nuclei are then assessed for immunofluorescence of NeuN to identify neuronal nuclei. C, Overlay of 2.0-µm PS bead gate (left) and neuronal nuclei (right) using FSC trigger on the Influx. Download Extended Data 1, TIF file. [file sup_enu-eN-MNT-0009-19-s01.tif]

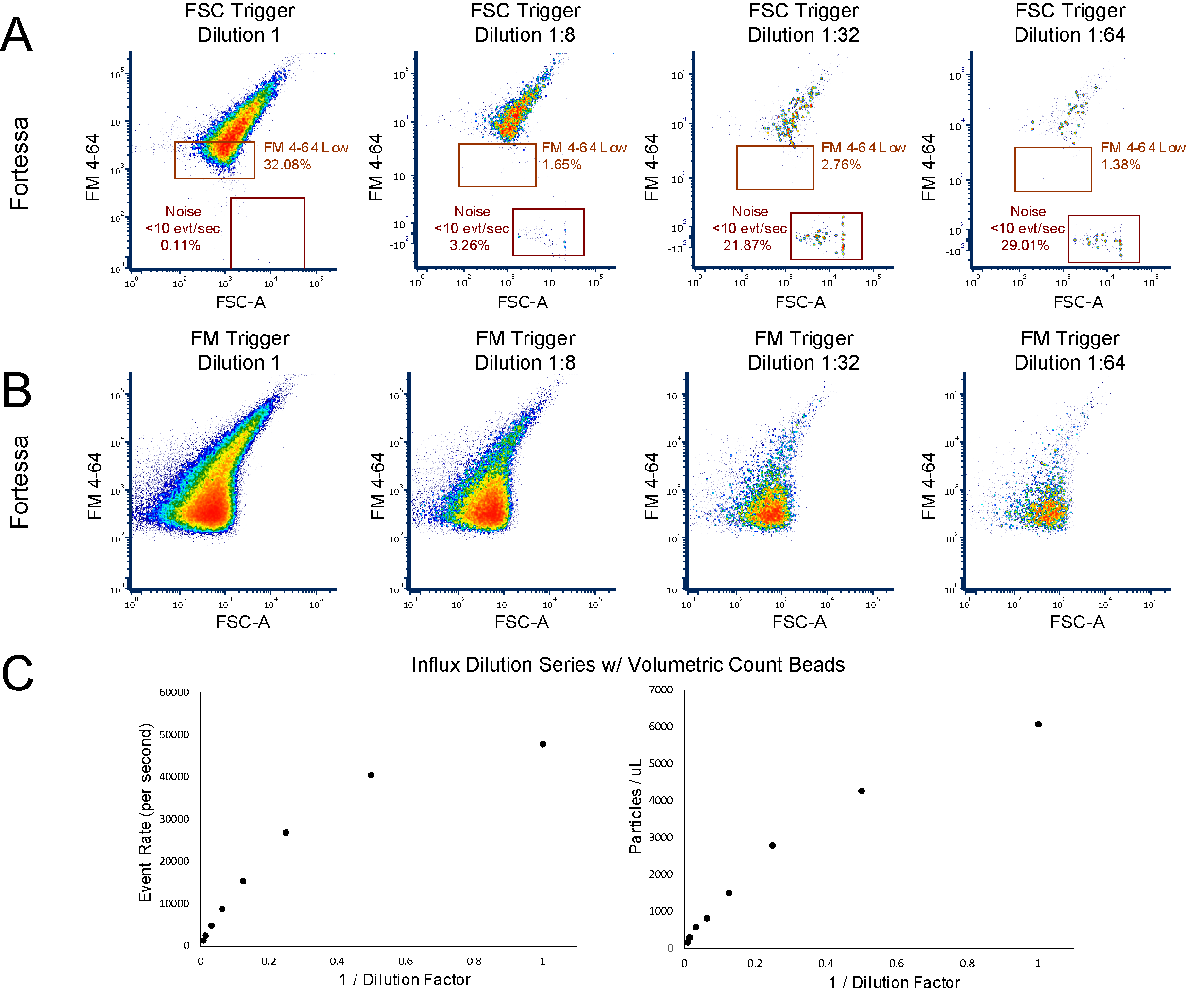

Supplement: Extended Data Figure 2-1 — Dilution series define a range of linear particle detection in P2 samples (Fortessa). A, Representative density plots of a P2 dilution series detected on the Fortessa in FSC-trigger mode. FM4-64 low gate was set arbitrarily to quantify disappearance of events in this region with dilution. Although FSC-trigger threshold was set to reduce noise to below 10 events/s, at the low end of the dilution curve these events begin to comprise a significant fraction of collected data. B, Representative density plots of same P2 dilution series as in A, but detected on the Fortessa in FM-trigger mode. C, Typical dilution series with FM4-64 triggering on the Influx, conducted with absolute counting beads. FM4-64 triggered event counts (excluding absolute counting beads) were used to determine particle concentration of each sample dilution. Event rate (left) and calculated particles/µl (right) are shown across the dilution series. Download Figure 2-1, TIF file. [file sup_enu-eN-MNT-0009-19-s02.tif]

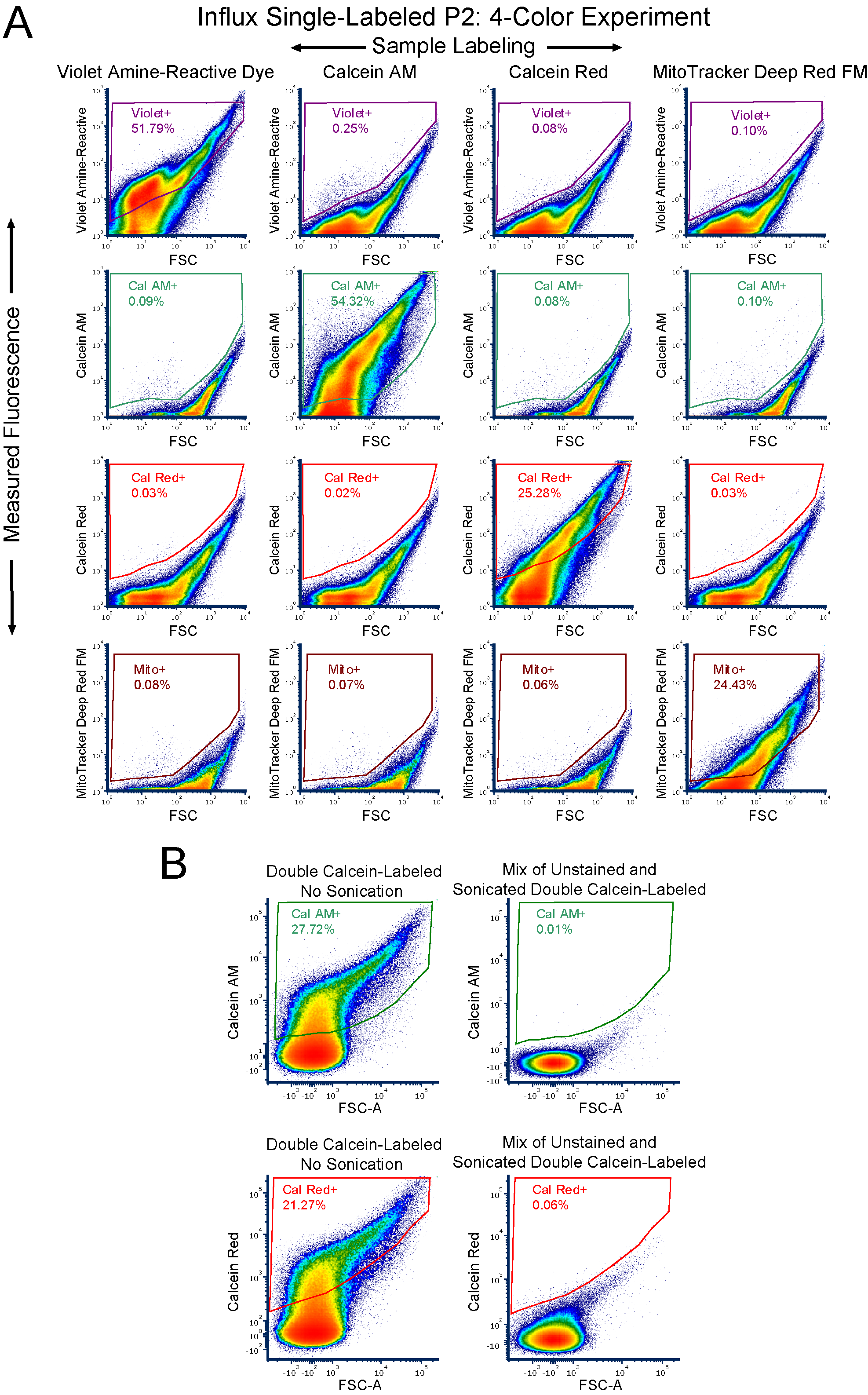

Supplement: Extended Data Figure 3-1 — Lack of spectral overlap between dyes and lack of calcein dye transfer to unstained samples. A, Matrix of representative density plots demonstrating lack of spectral overlap between any of the four dyes used in multicolor aggregation assays. Columns represent P2 samples single-labeled with the indicated dye, while rows indicate the measured fluorescence in the detector for the indicated dye. All samples were detected by FM triggering on the Influx. B, A single P2 sample was split into equal aliquots, one of which was left unstained, while the other was truly doubled-labeled with calcein-AM and calcein red. The double-labeled sample was then sonicated in the equivalent volume and concentration of PBS and subsequently mixed with the unstained sample as in the standard multicolor aggregation assay. A small aliquot of the double-labeled sample (right) and the sonicated double-labeled/unstained mixture (left) were detected using FM triggering on the Influx. As shown, sonication fully disrupted all double-labeled particles, and none of the released calcein dye was acquired by the unstained sample. Download Figure 3-1, TIF file. [file sup_enu-eN-MNT-0009-19-s03.tif]

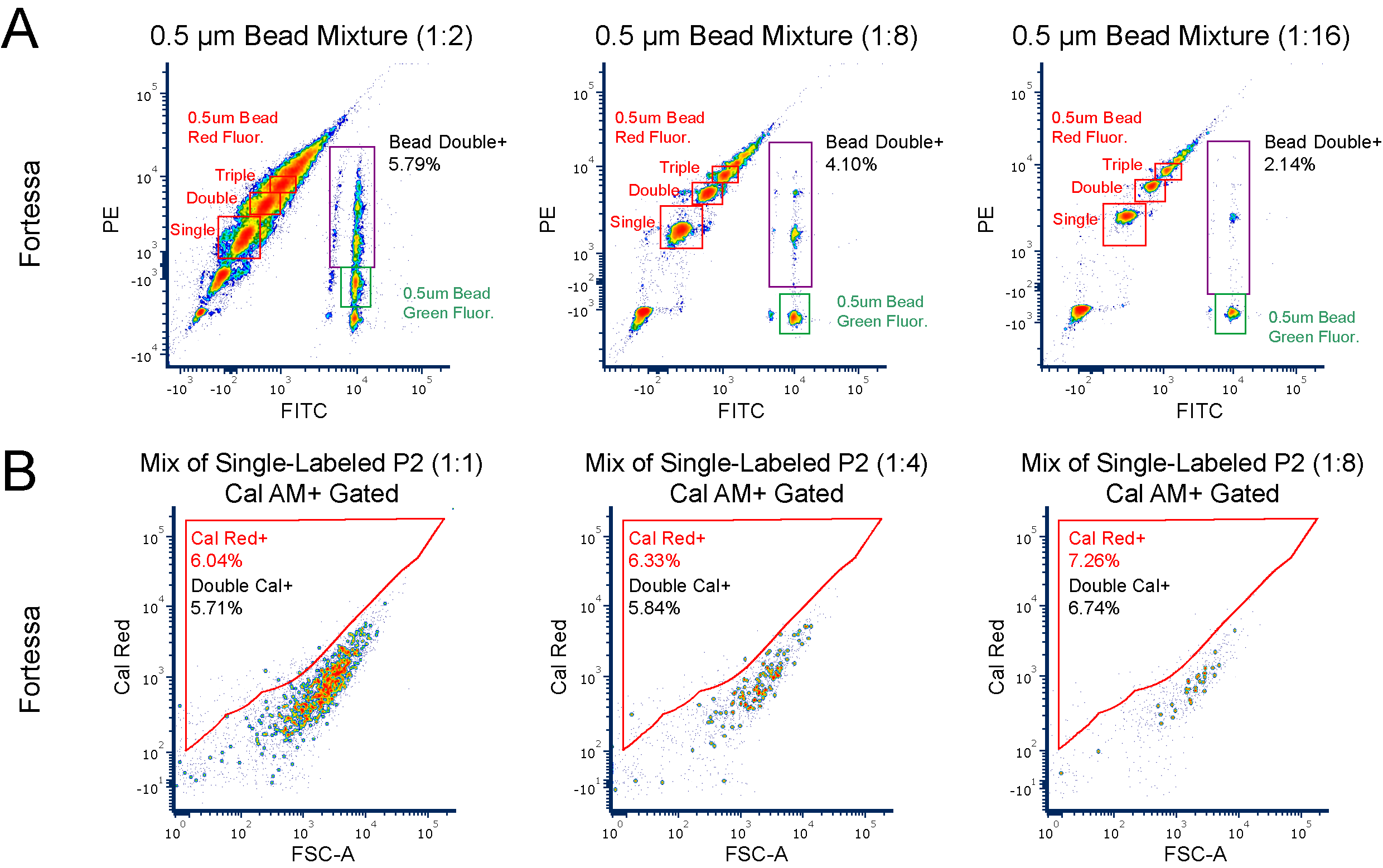

Supplement: Extended Data Figure 4-1 — Dilution series reduces false double-positive bead events but not double-calcein positive P2 events (Fortessa). A, Representative density plots of a dilution series for a mixture of fluorescent 0.5-µm PS beads detected using FSC triggering on the Fortessa. PS beads and double-positive events are distinguished by their green (FITC detector) or red (PE detector) fluorescence. In addition to clear overlaps between green and red fluorescent beads, we also observed what appeared to be doublet, triplet, and higher order multiplets of the Nile Red 0.5-µm PS beads. We confirmed this based on the doubling and tripling of the red fluorescence intensity of the “double” and “triple” gated populations relative to the single gate. At high concentrations (left), these multiplets could also be detected together with the green fluorescent beads. B, Representative density plots of a dilution series for a mixture of single calcein-labeled P2 samples detected using FM triggering on the Fortessa. Only events gated positive for calcein AM are displayed. Download Figure 4-1, TIF file. [file sup_enu-eN-MNT-0009-19-s04.tif]

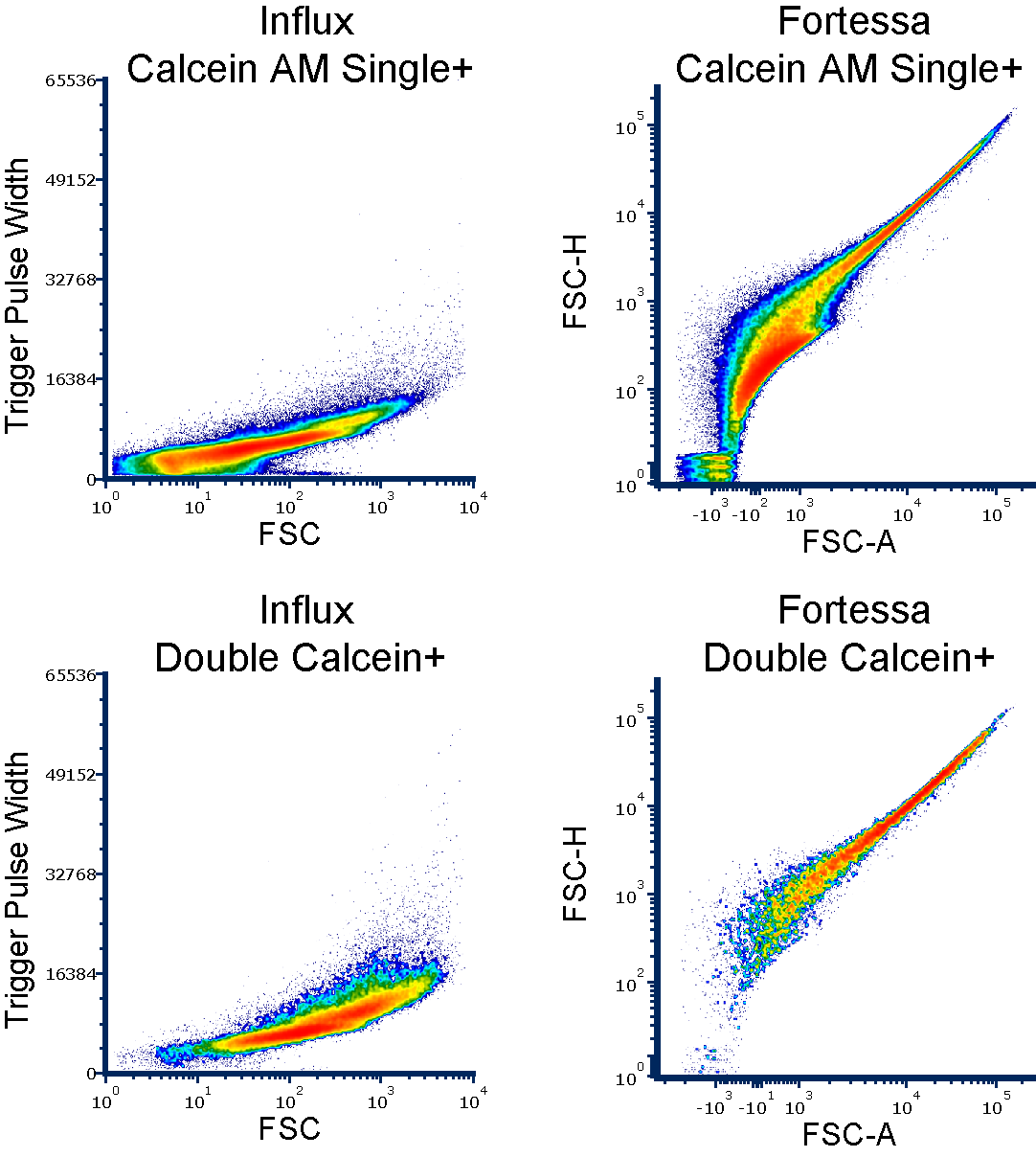

Supplement: Extended Data Figure 5-1 — Scatter-based singlet gating strategies employed in conventional flow cytometry are not effective for P2 samples. A mixture of single calcein-labeled P2 samples was analyzed by FM triggering on the Influx or the Fortessa. Only events gated for either single calcein-positive events (top) or double calcein-positive events (bottom) are displayed. Left, The Influx is an analog cytometer and does not measure height and width of pulses for the FSC detector; we instead compared the width of the trigger pulse (FM4-64) to the FSC intensity (equivalent to “area” on digital cytometers such as the Fortessa). Right, Comparison of area and height of FSC pulses on the Fortessa. On both the Influx and Fortessa, both single-positive and double-positive events display a singlet linear profile. Download Figure 5-1, TIF file. [file sup_enu-eN-MNT-0009-19-s05.tif]

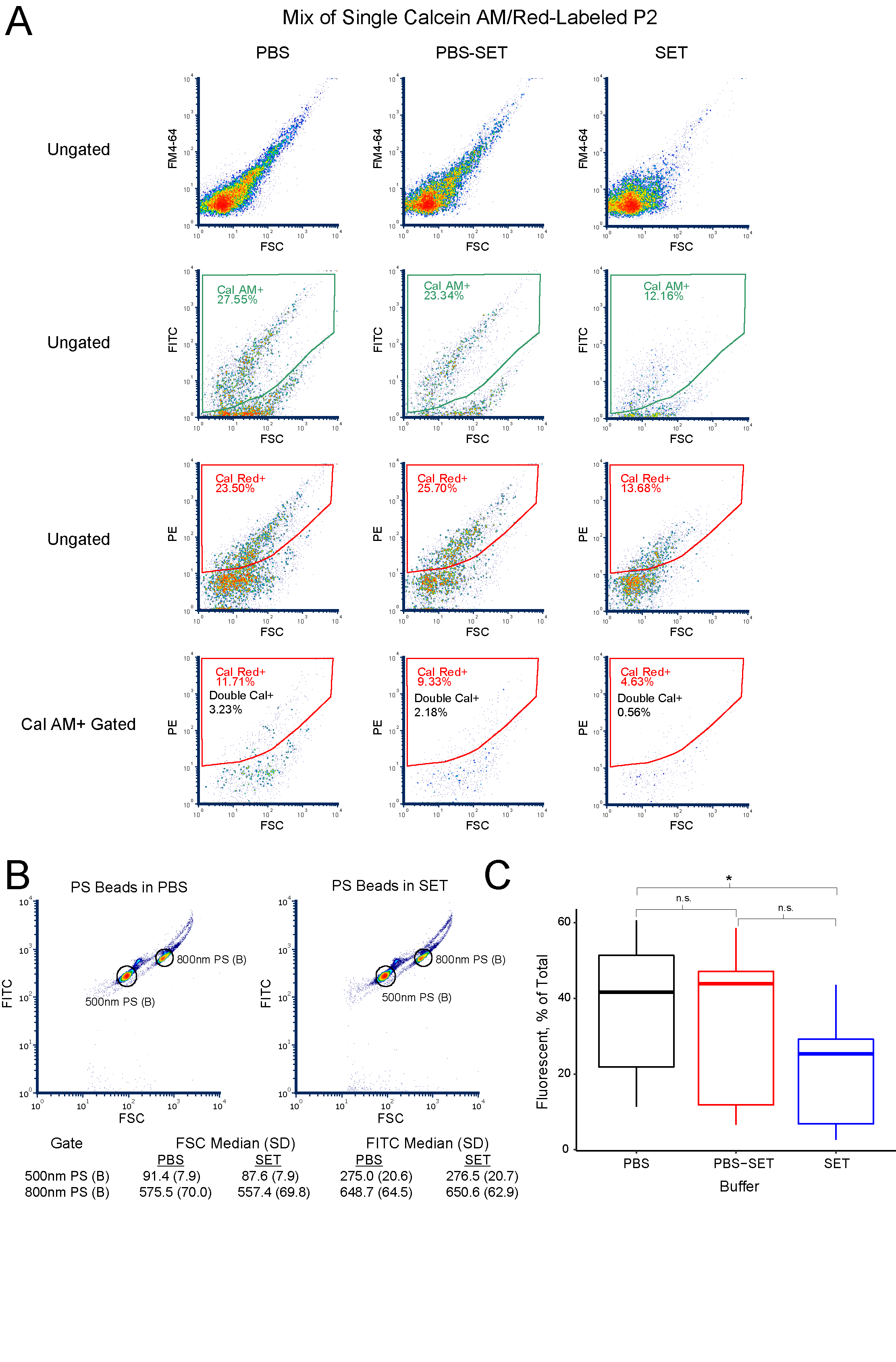

Supplement: Extended Data Figure 6-1 — Effects of different sample buffers on calcein labeling, FSC intensity, and fluorescence measurements. These experiments were conducted on the Influx using FSC triggering (for beads in B) or FM triggering (for P2 samples in A, C). A, Representative density plots for mixtures of single calcein-labeled P2 samples. Columns indicate the buffer designation for each sample, while the rows indicated the gated population displayed. B, FSC and green fluorescence (FITC detector) of fluorescent 0.5- and 0.8-µm PS beads (Bangs Labs; B) run in PBS (left) or SET (right) buffer. The median and SD of FSC and green fluorescence signal intensity are shown below for both gated bead populations. C, Box and whiskers plots of fluorescent events (all events positive for either calcein), expressed as a percentage of all events. Central bar represents the median. Lower and upper edges correspond to 25th and 75th percentiles. Lower and upper whiskers extend to the smallest or largest value no greater than 1.5 times the interquartile range away from the corresponding edge. Data are derived from the same single calcein-labeled P2 mixtures presented in Figure 6H (n = 10 for PBS, n = 8 for PBS-SET, and n = 10 for SET). A Student’s t test was performed comparing each buffer pair; *p < 0.05. Download Figure 6-1, TIF file. [file sup_enu-eN-MNT-0009-19-s06.tif]

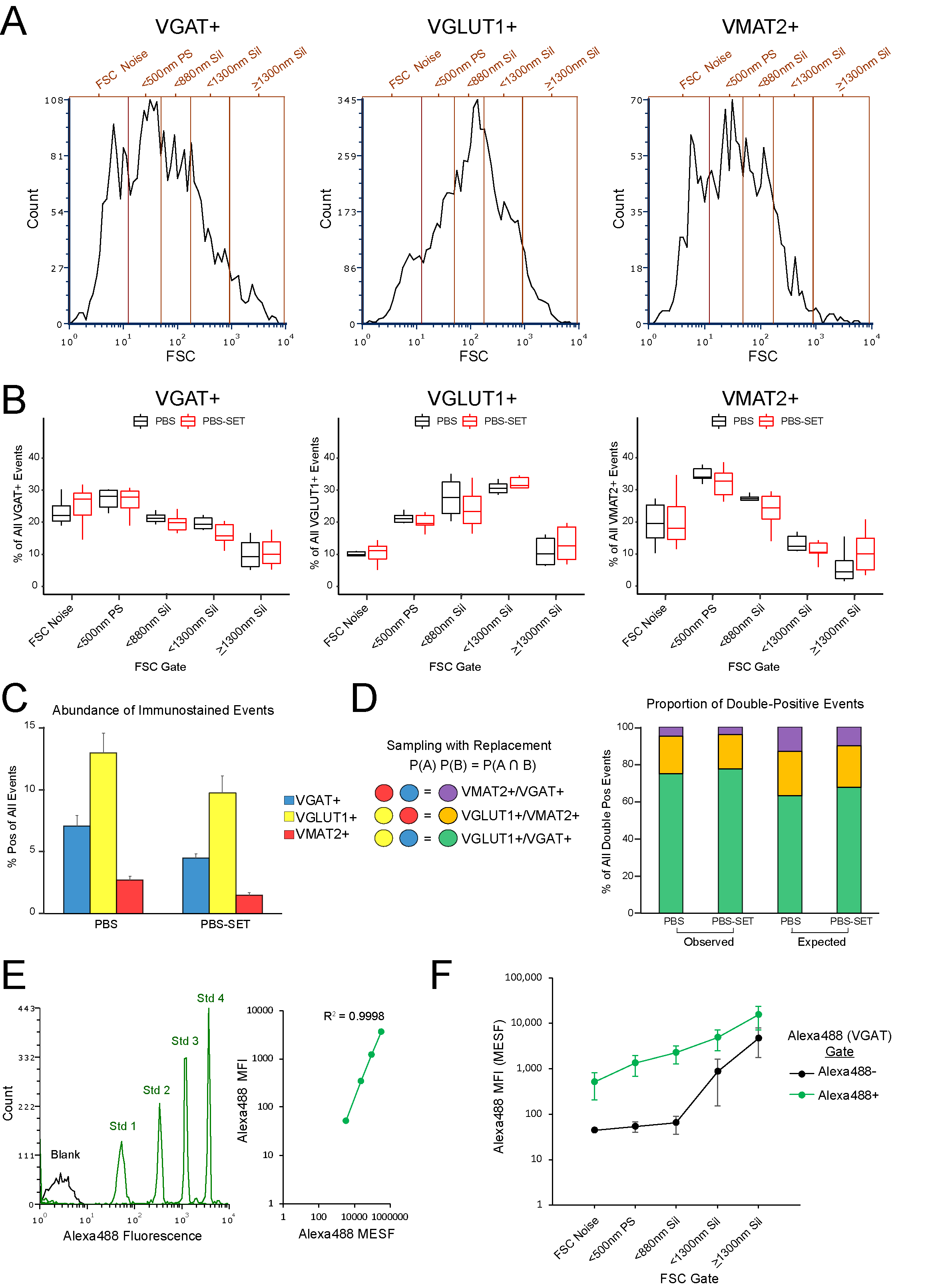

Supplement: Extended Data Figure 7-1 — Distribution of immunostained P2 events across FSC ranges and comparison between single-positive and double-positive event frequencies. All data are derived from the same samples as presented in Figure 7, i.e., P2 samples immunostained in PBS for VGAT, VGLUT1, and VMAT2 and run on the Influx with FM triggering in PBS or SET as sample buffer. A, Representative histogram with overlay of FSC gates. Only events gated as positive for VGAT (left), VGLUT1 (middle), or VMAT2 (right) are displayed. B, Box and whiskers plots of immunopositive events within each FSC gate, expressed as a percentage of all immunopositive events for the indicated antigen. Analogous to the histograms above, the sum across FSC gates is 100% for any one sample. Central bar represents the median. Lower and upper edges correspond to 25th and 75th percentiles. Lower and upper whiskers extend to the smallest or largest value no greater than 1.5 times the interquartile range away from the corresponding edge. C, Relative abundance of all events immunopositive for a given antigen, expressed as a percentage of all events. Data for are plotted as mean ± SEM for PBS or PBS-SET (n = 6 for each). D, left, A simple model for collisions of single-positive events leading to double-positive events is given by sampling with replacement. The relative abundance of any given double-positive event is estimated by multiplying the relative abundances of the two single-positive events. Right, Comparison of the observed and expected frequency for each type of double-positive event, expressed as a percentage of all double-positive events. Observed data represent the average for PBS or PBS-SET (n = 6 for each). E, Alexa Fluor 488 MESF calibration bead standards (left) used to construct a calibration curve based on measured median fluorescence intensity and the manufacturer’s stated MESF for each bead (right). F, A P2 sample stained for VGAT (with anti-mouse Alexa Fluor 488 secondary) was gated for immunonegative and immunopos [file sup_enu-eN-MNT-0009-19-s07.tif]

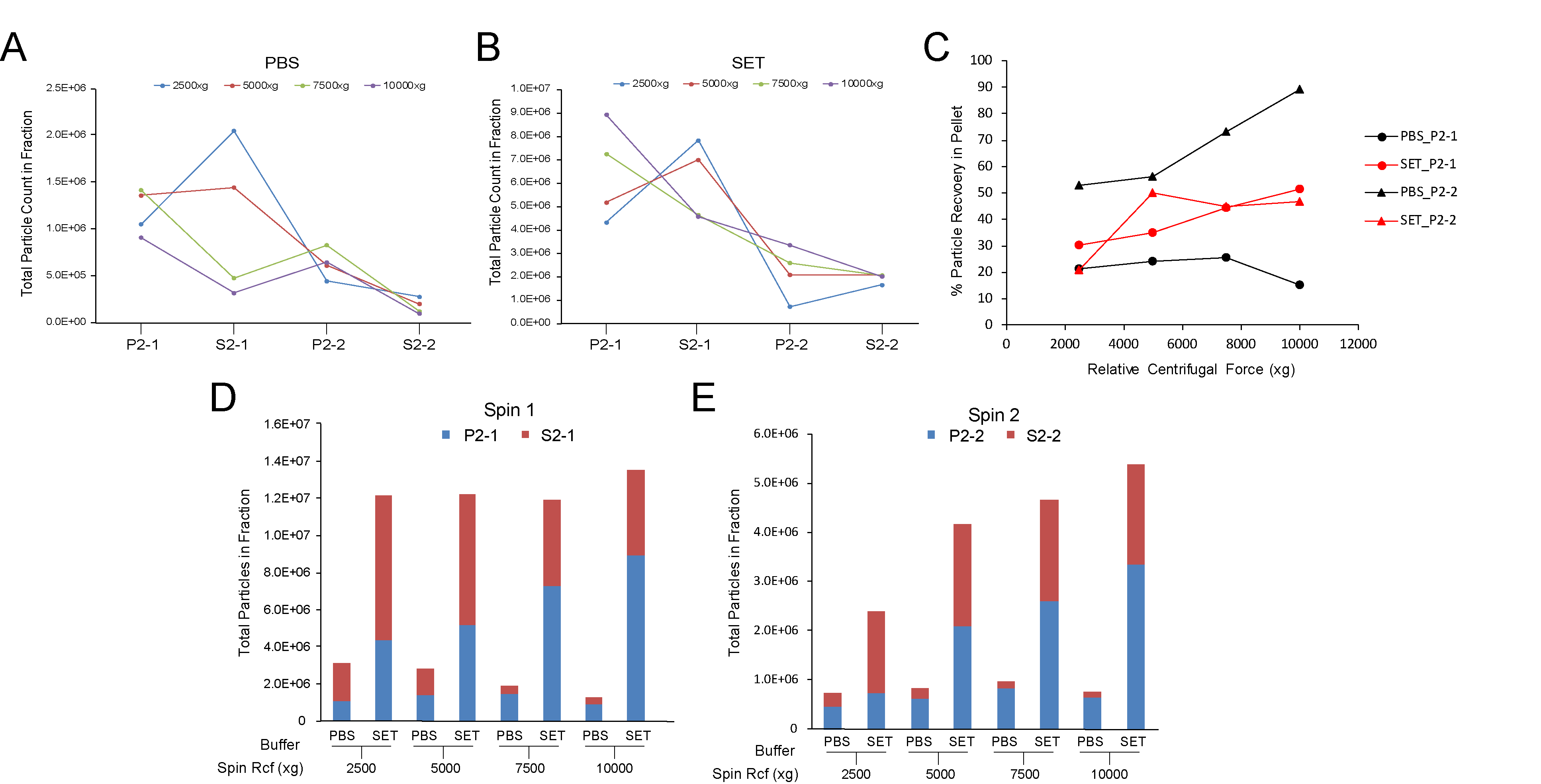

Supplement: Extended Data Figure 8-1 — Particle recovery in P2 samples following repeated centrifugations. All data are derived from the samples presented in Figure 8, i.e., P2 samples subjected to two centrifugations for 5 min in PBS or SET buffer at varying relative centrifugal force (× g). Absolute counting beads were added to each sample to determine absolute particle counts. A, Total particle counts in each fraction for P2-1, S2-1, P2-2, and S2-2 samples in PBS. B, Same as A but for SET buffer. C, Percent recovery in the pellet following each centrifugation at indicated rcf (× g). D, Total particle counts for pellets and supernatants across varying centrifugation rcf (× g) and in PBS or SET buffer for spin 1. E, Same as D but for spin 2. Download Figure 8-1, TIF file. [file sup_enu-eN-MNT-0009-19-s08.tif]
