# Supplementary material for: Critical Analysis of Particle Detection Artifacts in Synaptosome Flow Cytometry
Source: eNeuro. 2019 Jun 4;6(3):ENEURO.0009-19.2019. doi: 10.1523/ENEURO.0009-19.2019 (PMC6565374; doi:10.1523/ENEURO.0009-19.2019)
Supplement: Extended Data Figure 1-2 — FSC and SSC Measurements of PS and Silica Beads on Influx and Fortessa. Median FSC and SSC measurements with coefficient of variation (CV) for gated bead populations in Figure 1. Data are listed in order of ascending median FSC, which does not correspond to true size due to the refractive index mismatch between silica and PS beads. Download Figure 1-2, DOC file. [file sup_enu-eN-MNT-0009-19-s09.doc]

Figure 1-2: FSC and SSC Measurements of PS and Silica Beads on Influx and Fortessa

| Influx | | | |
| --- | --- | --- | --- |
| Gate | FSC Median (CV) | SSC Median (CV) | FITC Median |
| 585nm Si (A) | 38.3 (21.6) | 21.0 (91.8) | 1.0 |
| 500 nm PS singlet (A) | 77.0 (9.0) | 53.2 (18.7) | 2745.0 |
| 500 nm PS (B) | 104.4 (7.1) | 84.3 (12.3) | 266.6 |
| 500 nm PS doublet (A) | 175.4 (6.3) | 124.7 (28.0) | 4924.7 |
| 880 nm Si (A) | 254.6 (15.6) | 37.9 (75.9) | 1.0 |
| 800 nm PS (B) | 653.6 (8.7) | 214.1 (12.5) | 632.8 |
| 1300 nm Si (A) | 1286.8 (5.4) | 112.9 (46.6) | 1.0 |
| Fortessa | | | |
| Gate | FSC Median (CV) | SSC Median (CV) | FITC Median |
| 500 nm PS doublet (A) | 765.1 (53.3) | 4803.3 (14.6) | 86606.96 |
| 880 nm Si (A) | 943.8 (49.7) | 1077.3 (13.6) | -71.76 |
| 500 nm PS singlet (A) | 1022.5 (25.0) | 2356.1 (24.9) | 46027.60 |
| 1300 nm Si (A) | 8247.9 (10.9) | 3283.9 (4.7) | -96.60 |

Median FSC and SSC measurements with coefficient of variation (CV) for gated bead populations in Figure 1. Data are listed in order of ascending median FSC, which does not correspond to true size due to the refractive index mismatch between silica and PS beads.
