# Supplementary material for: Critical Analysis of Particle Detection Artifacts in Synaptosome Flow Cytometry
Source: eNeuro. 2019 Jun 4;6(3):ENEURO.0009-19.2019. doi: 10.1523/ENEURO.0009-19.2019 (PMC6565374; doi:10.1523/ENEURO.0009-19.2019)
Supplement: Extended Data Figure 7-2 — Single-, Double-, and Triple-Positive Percentages for Each Type of Immunopositive Event. Data derived from samples shown in Fig. 7B-C (Mean ± SEM for n=6 replicates each of buffer). Download Figure 7-2, DOC file. [file sup_enu-eN-MNT-0009-19-s10.doc]

Figure 7-2: Single-, Double-, and Triple-Positive Percentages for Each Type of Immunopositive Event

| VGAT+ | | | | | | |
| --- | --- | --- | --- | --- | --- | --- |
| FSC Gate | Single-Positive Events,  % of all VGAT+ Events | | Double-Positive Events,  % of all VGAT+ Events | | Triple-Positive Events,  % of all VGAT+ Events | |
|  | PBS | PBS-SET | PBS | PBS-SET | PBS | PBS-SET |
| All Sizes | 56 ± 4 | 60 ± 4 | 33 ± 3 | 31 ± 2 | 10 ± 2 | 9 ± 2 |
| FSC Noise | 80 ± 5 | 83 ± 5 | 13 ± 2 | 11 ± 3 | 7 ± 3 | 5 ± 3 |
| <500nm PS | 68 ± 4 | 71 ± 5 | 21 ± 3 | 20 ± 2 | 11 ± 3 | 9 ± 3 |
| <880nm Sil | 57 ± 4 | 64 ± 5 | 31 ± 4 | 29 ± 4 | 11 ± 2 | 7 ± 2 |
| <1300nm Sil | 35 ± 5 | 37 ± 4 | 57 ± 4 | 56 ± 4 | 8 ± 1 | 6 ± 3 |
| >1300nm Sil | 12 ± 3 | 7 ± 2 | 73 ± 2 | 71 ± 4 | 15 ± 3 | 22 ± 2 |
| VGLUT1+ | | | | | | |
| FSC Gate | Single-Positive Events,  % of all VGLUT1+ Events | | Double-Positive Events,  % of all VGLUT1+ Events | | Triple-Positive Events,  % of all VGLUT1+ Events | |
|  | PBS | PBS-SET | PBS | PBS-SET | PBS | PBS-SET |
| All Sizes | 71 ± 3 | 78 ± 2 | 23 ± 3 | 18 ± 2 | 5 ± 1 | 4 ± 0 |
| FSC Noise | 75 ± 6 | 79 ± 5 | 17 ± 3 | 15 ± 3 | 8 ± 3 | 6 ± 2 |
| <500nm PS | 73 ± 2 | 79 ± 2 | 20 ± 1 | 16 ± 1 | 7 ± 2 | 5 ± 1 |
| <880nm Sil | 76 ± 3 | 83 ± 2 | 20 ± 3 | 15 ± 1 | 4 ± 0 | 3 ± 0 |
| <1300nm Sil | 73 ± 5 | 82 ± 2 | 24 ± 4 | 16 ± 2 | 3 ± 1 | 2 ± 1 |
| >1300nm Sil | 51 ± 8 | 61 ± 5 | 40 ± 6 | 31 ± 4 | 9 ± 2 | 9 ± 1 |
| VMAT2+ | | | | | | |
| FSC Gate | Single-Positive Events,  % of all VMAT2+ Events | | Double-Positive Events,  % of all VMAT2+ Events | | Triple-Positive Events,  % of all VMAT2+ Events | |
|  | PBS | PBS-SET | PBS | PBS-SET | PBS | PBS-SET |
| All Sizes | 46 ± 8 | 48 ± 7 | 28 ± 6 | 27 ± 4 | 26 ± 3 | 26 ± 3 |
| FSC Noise | 63 ± 9 | 64 ± 5 | 21 ± 4 | 23 ± 2 | 16 ± 6 | 14 ± 5 |
| <500nm PS | 53 ± 10 | 58 ± 9 | 27 ± 6 | 22 ± 4 | 21 ± 5 | 20 ± 6 |
| <880nm Sil | 44 ± 11 | 48 ± 11 | 34 ± 9 | 33 ± 7 | 22 ± 3 | 19 ± 4 |
| <1300nm Sil | 32 ± 6 | 47 ± 13 | 39 ± 7 | 34 ± 9 | 29 ± 4 | 20 ± 7 |
| >1300nm Sil | 9 ± 4 | 1 ± 1 | 14 ± 5 | 22 ± 3 | 78 ± 6 | 76 ± 3 |

Data derived from samples shown in Fig. 7B-C (Mean ± SEM for n=6 replicates each of buffer)
