# Supplementary material for: Critical Analysis of Particle Detection Artifacts in Synaptosome Flow Cytometry
Source: eNeuro. 2019 Jun 4;6(3):ENEURO.0009-19.2019. doi: 10.1523/ENEURO.0009-19.2019 (PMC6565374; doi:10.1523/ENEURO.0009-19.2019)
Supplement: Extended Data Figure 7-3 — MESF Calibration of AlexaFluor488 Immunostaining. Numerical data corresponding to those shown in Figure 7-1E-F. Download Figure 7-3, DOC file. [file sup_enu-eN-MNT-0009-19-s11.doc]

Figure 7-3: MESF Calibration of AlexaFluor488 Immunostaining

| Sample_FluorGate | Size Gate | Median Fluorescence Intensity (MESF) | BD-rSD (MESF) |
| --- | --- | --- | --- |
| MESF-Blank | N/A |  | 46 |
| MESF-Std1 | N/A | 3226 | 355 |
| MESF-Std2 | N/A | 22646 | 1693 |
| MESF-Std3 | N/A | 97603 | 4332 |
| MESF-Std4 | N/A | 336257 | 11637 |
| MsVGAT_Alexa488- | FSC Noise | 43 | 1 |
| MsVGAT_Alexa488- | <500nm PS | 54 | 14 |
| MsVGAT_Alexa488- | <880nm Sil | 64 | 28 |
| MsVGAT_Alexa488- | <1300nm Sil | 869 | 718 |
| MsVGAT_Alexa488- | >1300nm Sil | 4682 | 2944 |
| MsVGAT_Alexa488+ | FSC Noise | 513 | 312 |
| MsVGAT_Alexa488+ | <500nm PS | 1310 | 644 |
| MsVGAT_Alexa488+ | <880nm Sil | 2204 | 956 |
| MsVGAT_Alexa488+ | <1300nm Sil | 4827 | 2330 |
| MsVGAT_Alexa488+ | >1300nm Sil | 14931 | 8041 |

Numerical data corresponding to those shown in Figure 7-1E-F.
